# Supplementary material for: Benefits and harms of perioperative high fraction inspired oxygen for surgical site infection prevention: a protocol for a systematic review and meta-analysis of individual patient data of randomised controlled trials
Source: BMJ Open. 2023 Oct 29;13(10):e067243. doi: 10.1136/bmjopen-2022-067243 (PMC10619062; doi:10.1136/bmjopen-2022-067243)
Supplement: Author's manuscript [file bmjopen-2022-067243.draft_revisions.pdf]

BMJ Open is committed to open peer review. As part of this commitment we make the peer review history of every article we publish publicly available.

When an article is published we post the peer reviewers' comments and the authors' responses online. We also post the versions of the paper that were used during peer review. These are the versions that the peer review comments apply to.

The versions of the paper that follow are the versions that were submitted during the peer review process. They are not the versions of record or the final published versions. They should not be cited or distributed as the published version of this manuscript.

BMJ Open is an open access journal and the full, final, typeset and author-corrected version of record of the manuscript is available on our site with no access controls, subscription charges or pay-per-view fees (http://bmjopen.bmj.com).

If you have any questions on BMJ Open's open peer review process please email info.bmjopen@bmj.com

BMJ Open

The benefits and harms of perioperative high fraction inspired oxygen for surgical site infection prevention: a protocol for a systematic review and meta-analysis of individual patient data of randomised controlled trials

Journal:	*BMJ Open*
Manuscript ID	bmjopen-2022-067243
Article Type:	Protocol
Date Submitted by the Author:	15-Aug-2022

Complete List of Authors:	de Jonge, Stijn; Amsterdam UMC Locatie AMC, Surgery; Amsterdam UMC Locatie AMC, Anaesthesiology<br>Hulskes, Rick; Amsterdam UMC Locatie AMC, Anaesthesiology; Amsterdam UMC Locatie AMC, Surgery<br>Weenink, Robert ; Amsterdam UMC Locatie AMC, Anaesthesiology<br>Meyhoff, Christian; Copenhagen University Hospital, Anaesthesia and Intensive Care<br>Leslie, Kate; The Royal Melbourne Hospital, Anaesthesia<br>Myles, Paul; Monash University, Anaesthesia and Perioperative Medicine<br>Forbes, Andrew; Monash University, Epidemiology and Preventive Medicine<br>Greif, Robert ; Inselspital Universitatsspital Bern, Anaesthesiology and PAin Therapy<br>Akca, Ozan; Johns Hopkins University, Anaesthesiology & Critical Care Medicine<br>Kurz, Andrea; Cleveland Clinic, General Anesthesiology<br>Sessler, Daniel; Cleveland Clinic,<br>Martin, Janet; University of Western Ontario, Anaesthesiology & Perioperative Medicine and Department of Epidemiology & Biostatistics<br>Dijkgraaf, Marcel; Amsterdam UMC Locatie AMC, Epidemiology and Data Science<br>Pryor, Kane; Weill Cornell Medical College, Anesthesiology<br>Belda, F. Javier; Hospital Clinico Universitario, Surgery; Hospital Clinico Universitario, Anaesthesia and Critical Care<br>Ferrando, Carlos; Hospital Clinico Universitario, Anesthesiology and Cirtical Care<br>Gurman, Gabriel; Ben-Gurion University of the Negev, Anaesthesiology and Critical Care Medicine<br>Scifres, Christina; Indiana University School of Medicine,<br>McKenna, David; Wright State University, Obstetrics and Gynaecology<br>Chan, MTV; The Chinese University of Hong Kong, Anesthesia and Intensive Care<br>Thibon, Pascal; Centre d'appui pour la prevention des infections associees aux soins d'Ile-de-France<br>Mellin-Olsen, Jannicke; World Federation of Societies of Anesthesiologists<br>Allegranzi, B; World Health Organization<br>Boermeester, Marja; Amsterdam UMC Locatie AMC, Surgery<br>Hollmann, Markus; Amsterdam UMC Locatie AMC, Anaesthesiology

Keywords:	ANAESTHETICS, WOUND MANAGEMENT, Infection control < INFECTIOUS DISEASES, SURGERY

SCHOLARONE™ Manuscripts

TITLE PAGE

The benefits and harms of perioperative high fraction inspired oxygen for surgical site infection prevention: a protocol for a systematic review and meta-analysis of individual patient data of randomised controlled trials

S. W. de Jonge[1a,b,2*], R. H. Hulskes[1a,2], R. P. Weenink[2], C. S. Meyhoff[3], K. Leslie[4], P. S. Myles[5], A. Forbes[6], R. Greif[7,8], O. Akça[9], A. Kurz[10], D. I. Sessler[11], J. Martin[12], M. G. Dijkgraaf[13a,b], K. O. Pryor[14], F. J. Belda[15], C. Ferrando[16a,16b], G. M. Gurman[17], C. Scifres[18], D. S. McKenna[19], M. T. V. Chan[20], P. Thibon[21], J. Mellin-Olsen[22], B. A. Allegranzi[23], M. A. Boermeester[1a, b], M. W. Hollmann[2]

[1a] Department of Surgery, Amsterdam UMC, University of Amsterdam, Amsterdam, The Netherlands
[1b] Gastroenterology Endocrinology & Metabolism, Amsterdam, the Netherlands, Amsterdam, The Netherlands
[2] Department of Anaesthesiology, Amsterdam UMC, University of Amsterdam, Amsterdam, The Netherlands
[3] Department of Anaesthesia and Intensive Care, Copenhagen University Hospital – Bispebjerg and Frederiksberg, Copenhagen, Denmark
[4] Department of Critical Care, Melbourne Medical School, University of Melbourne, Australia
[5] Department of Anaesthesiology and Perioperative Medicine, Alfred Hospital and Monash University, Melbourne, Australia
[6] Department of Epidemiology and Preventive Medicine, Monash University, Melbourne, Australia
[7] Department of Anaesthesiology and Pain Medicine, Bern University Hospital, University of Bern, Bern, Switzerland
[8] School of Medicine, Sigmund Freud University Vienna, Vienna, Austria
[9] Department of Anaesthesiology & Critical Care Medicine, Johns Hopkins University, Maryland, United States of America
[10] Department of Anaesthesiology and department of Outcomes Research, Cleveland Clinic Cleveland Ohio, Ohio, United States of America
[11] Department of Outcomes Research, Cleveland Clinic Cleveland Ohio, Ohio, United States of America
[12] Department of Anaesthesiology & Perioperative Medicine and Department of Epidemiology & Biostatistics, University of Western Ontario, Canada
[13a] Amsterdam UMC location University of Amsterdam, Epidemiology and Data Science, Amsterdam, The Netherlands
[13b] Amsterdam Public Health, Methodology, Amsterdam, The Netherlands
[14] Department of Anaesthesiology, Weil Medical College of Cornell University, New York City, New York, United States of America
[15] Department of Surgery and Department of Anaesthesia Critical Care, Hospital Clinico Universitario De Valencia, Valencia, Spain
[16a] Department of Anaesthesiology and Critical Care, Hospital Clínic I Provincial, Barcelona, Spain
[16b] CIBER de Enfermedades Respiratorias, Instituto de Salud Carlos III, Madrid, Spain
[17] Department of Anaesthesiology and Critical Care Medicine, Ben Gurion University of the Negev, Beer Sheva, Israel
[18] Department of Obstetrics and Gynaecology, Indiana University School of Medicine, Indianapolis, United States of America
[19] Department of Obstetrics and Gynaecology, Wright State University and Miami Valley Hospital, Ohio, United States of America
[20] Department of Anaesthesia and Intensive Care, Chinese University of Hong Kong, Hong Kong Special Administrative Region, China
[21] Centre d'appui pour la Prévention des Infections Associées aux Soins, CPias Normandie, Centre Hospitalo-Universitaire, Caen, Normandy, France
[22] World Federation of Societies of Anaesthesiologists
[23] World Health Organization, Service Delivery and Safety, Infection Prevention and Control Global Unit

***Corresponding author**

Corresponding author: S. W. de Jonge, Amsterdam UMC, University of Amsterdam, Meibergdreef 9, 1105 AZ Amsterdam, the Netherlands, email: s.w.dejonge@amsterdamumc.nl, telephone number 0031 (0)20 566 9111.

Word count: 2652 (excluding title page, abstract, article summary, tables, ethics and dissemination, and references)

Keywords: general anaesthesia; hyperoxia; postoperative outcome; surgical site infection; surgical wound infection

For peer review only

For peer review only - http://bmjopen.bmj.com/site/about/guidelines.xhtml

ABSTRACT

Introduction: The use of high fraction of inspired oxygen (FiO_2) intraoperatively for the prevention of surgical site infection (SSI) remains controversial. Consequently, there is considerable practice variation in oxygen use. Early promising results have been replicated with varying success, and subsequent meta-analyses are equivocal. Since the initial promising results, perioperative care has changed considerably with consequences for hemodynamics, microcirculation, and peripheral oxygen delivery. These changes may explain the inconsistency in results, but the available published data provides insufficient detail on the participant level to test this hypothesis. The purpose of this individual participant data meta-analysis is to assess the described benefits and harms of intraoperative high (0.60-1.00) FiO_2 compared to regular (0.21-0.40) FiO_2 and its potential effect modifiers.

Methods and analysis: The initial search conducted for the WHO guidelines for the prevention of surgical site infection reviews will be updated. Medical databases and online trial registries will be searched to include all randomised and quasi-randomised controlled trials comparing the effect of intraoperative high FiO_2 (0.60-1.00) to regular FiO_2 (0.21-0.40) in patients undergoing surgery. Two researchers will independently assess articles retrieved by the search against the eligibility criteria for inclusion and methodological quality. Investigators of the identified trials will be invited to collaborate, comment on the study protocol, and supply the individual participant data of their initial trial and any additional follow-up data. The primary outcomes will be SSI within 90 days after surgery by the author's discretion, serious adverse events, and all-cause mortality within the longest available follow-up. Data will be analysed with the one-step approach. Additional analysis included exploration of effect-modifiers. The certainty of evidence will be assessed using GRADE methodology.

Ethics and dissemination: Ethics approval is not required. Investigators will de-identify individual participant data before it is shared. The results will be submitted to a peer-review journal.

Trial Registration Number: PROSPERO CRD42018090261

For peer review only - http://bmjopen.bmj.com/site/about/guidelines.xhtml

ARTICLE SUMMARY

Strengths and limitations of this study

- Individual participant data meta-analysis (IPD MA) of (quasi-)randomised controlled trials is considered the gold standard of evidence-based medicine, providing the best possible analysis of the available data on the participant level, permitting the investigation of potential effect modifiers.
- IPD MA requires the collaboration of all investigators that have published data on the relevant topic and leads to a broad consensus on the outcome and interpretation of the analysis
- IPD MA depends on the quality of data that is made available by the authors of the original studies.

For peer review only

For peer review only - http://bmjopen.bmj.com/site/about/guidelines.xhtml

INTRODUCTION

Surgical site infection (SSI) is one of the most common healthcare-associated infections and leads to morbidity, mortality, and longer hospital stay.(1-4) The attributable costs can be more than €14.000 per SSI, and European totals are estimated to range from €1.5 to €19 billion per year.(5, 6) In 2016-2017, both the World Health Organization (WHO) and the Centres of Disease Control and Prevention (CDC) independently released evidence-based guidelines on the prevention of SSI that included a recommendation in favour of the administration of high fraction of inspired (FiO_2) for patients undergoing surgery under general anaesthesia.(7-9) This has led to a debate between opponents and proponents of the use of high FiO_2 in several editorials and correspondences across medical speciality literature.(10-20) Concerns were raised on the safety of the use of high FiO_2 as well as the different study results in supporting and not supporting use of high FiO_2 to reduce SSI.(10-20)

In response to these concerns, the WHO conducted an independent systematic review on the safety of high intraoperative FiO_2 and updated the systematic review on its effectiveness, excluding the disputed trials.(21, 22) No evidence of harm to discourage the use of high FiO_2 was found, yet the evidence of an effect of SSI had become weaker, and the recommendation was adjusted accordingly.(23) Despite many randomised controlled trials, various meta-analyses, and guideline recommendations, uncertainty remains. This leads to practice variation that inevitably exposes patients to suboptimal care.(24) There is a need for better understanding and consensus on this issue.

Since the early promising results, perioperative care has changed considerably. Open abdominal surgery has been largely replaced by laparoscopic surgery, fluid management has moved from liberal to restrictive, to advanced goal directed regimens and active perioperative warming has become a mainstay.(25-27) All these changes have considerable consequences for hemodynamic, microcirculation, and eventually peripheral oxygen delivery.(28-30) These changes may explain the inconsistency in reproducibility, but the available data provides insufficient detail on the participant level to test the potential of high FiO_2. Meta-analysis of individual participant data uses the raw individual-level data from the original study for synthesis and overcomes this limitation.(31, 32) IPD MA enables analysis of uniform outcomes with more statistical power and assessment of potential effect modifiers.(33, 34) Importantly, IPD MA requires collaboration with all published researchers on the topic leading to a broad consensus on the outcome of data analysis and interpretation.

The purpose of this IPD MA is to assess the potential benefits and harms of intraoperative high (0.60-1.00) FiO_2 compared to traditional (0.21-0.40) FiO_2 and its effect modifiers in patients undergoing surgery. This IPD MA is initiated by the University of Amsterdam / Amsterdam University Medical

For peer review only - http://bmjopen.bmj.com/site/about/guidelines.xhtml

Center, and encouraged by the WHO and the World Federation of Societies of Anaesthesiologists (WFSA) to provide patients and practitioners with the best possible evidence and guidance on this disputed area and will give clearance of the disputed hypothesis that high FiO_2 reduces the incidence of SSI.

For peer review only

For peer review only - http://bmjopen.bmj.com/site/about/guidelines.xhtml

METHODS AND ANALYSIS

Protocol and registration

This study protocol is registered with the International Prospective Register of Systematic Reviews (PROSPERO) on 7 March 2018 and was last updated on 15 July 2022 (registration number CRD42018090261). The study protocol is designed and written to adhere to the Preferred Reporting Items for Systematic Review and Meta-Analysis Protocols (PRISMA-P)(35) and the Preferred Reporting Items for Systematic Review Meta-Analysis of individual patient data (IPD) (PRISMA-IPD).(32)

Patient and Public Involvement Statement

This project is encouraged by the World Health Organization (WHO), and the World Federation of Societies of Anesthesiologists (WFSA) to provide patients and practitioners with the best possible evidence and guidance on this disputed area. WHO and WFSA have provided external independent review and advice on research direction and aim.

Governance

This study is an initiative of the Amsterdam University Medical Centre, encouraged by the WHO and the WFSA. Both organisations recognise the urgent need for this research and provide external independent review and advice. The writing committee consists of the study coordinator, two reviewers, a lead methodologist, and a principal investigator from both the surgery and the anaesthesiology department of the Amsterdam University Medical Centre and two external content matter experts. The writing committee is entirely independent of the initial trials and has full responsibility for all methodological decisions. A broader steering committee with representatives of the collaborating trial groups identified during the project will be invited to comment on and co-author the final protocol and IPD MA report. By sharing their IPD, collaborators will obtain one co-authorship on the IPD MA report and one additional co-authorship if data of more than 300 participants is shared. For transparency and against intellectual bias, a record will be kept of all comments. Any important amendments to the protocol will be recorded in PROSPERO record and discussed in the methods section of the final report.

Eligibility criteria

For peer review only - http://bmjopen.bmj.com/site/about/guidelines.xhtml

We will include all randomised and quasi-randomised controlled trials comparing the effect of intraoperative high FiO_2 (0.60-1.0) to traditional FiO_2 (0.21-0.40) in patients undergoing surgery. These trials may include patients of any age undergoing surgery except for neonates, regardless publication, language, or year of conduct and should include at least data on age, sex, mean FiO_2 administered, method of oxygen administration, SSI, mortality, or other serious adverse events. Any outcome found to be recorded in these trials will be included in the analysis. Studies without random or quasi-random treatment allocation, animal studies, and studies outside of the intraoperative period will be excluded.

Identifying studies – information sources

The initial search conducted for the WHO guideline will be updated by a professional information specialist.(21, 22) Medical databases will be searched, including MEDLINE, EMBASE, CENTRAL, CINHAL, and the WHO regional databases. Online trial registries will be searched to identify potential unpublished evidence or any ongoing trials. The search will not be limited by language or date of publication. A final update will be conducted before the final round of revisions preceding submission for publication. The reference list of all included studies will be hand searched for any additional relevant trials not already identified through database searching. All corresponding authors of relevant clinical trials will be contacted to review the list of identified studies for the omission of potentially relevant studies missed by the search.

Study selection process

Two reviewers will independently assess articles retrieved by the search against the eligibility criteria. After screening the title and abstract, the full text of potentially eligible papers will be retrieved and assessed. When no full paper exists, or trial eligibility is in doubt, the study authors will be contacted to provide further information. Any discrepancies in study selection will be resolved through consensus and discussion with a senior author. All studies that pass title and abstract screening but were not eligible for inclusion will be listed with the reasons for exclusion.

Study collaboration invitation

Authors of eligible studies will be contacted and invited to collaborate on the IPD MA. An email invitation will be sent to the corresponding authors outlining the IPMDA goals. If no reply is received within two weeks, a second email request will be sent to the corresponding and first author. If no

For peer review only - http://bmjopen.bmj.com/site/about/guidelines.xhtml

response is received again, we will try to contact all authors by email and telephone. IPD data will be considered unavailable if numerous times (at least five) no reply is received if authors no longer have access to the study data or consent to collaboration.

Data collection process

The collaborating investigators will be requested to sign a data transfer agreement describing the ownership and storage of the IPD before de-identified IPD is shared. Whenever possible, data collection, interview on the protocol, and formal handoff on the data codebook will be done electronically via email, videoconference, or a suitable alternative. Whenever requested by the original investigator, a researcher will visit the investigators for a physical data transfer, in-person interview, and data codebook handoff. In the unlikely event that individual patient data will not be made available, the reason will be recorded. The aggregate data of the study will be used in a sensitivity analysis. Aggregate data collection will be performed as appropriate for a regular meta-analysis by two independent reviewers according to a predefined data extraction sheet and overseen by a senior author to settle potential discrepancies. The University of Amsterdam's Clinical Research Unit will facilitate secure data storage.

Data items

Data items will include all data recorded by the initial trial investigators including, but not limited to the items listed in table 1 and table 2.

**Table 1. Baseline and procedure characteristics**	
Baseline	Sex, age, BMI (kg/m^2), ASA physical status score, smoking status, peripheral vascular disease, diabetes, (metastatic) cancer, congestive heart disease, (pulmonary) hypertension, chronic obstructive pulmonary disease, immunosuppressant use, peripheral oxygen saturation (%), glucose (mg/dL), indication for surgery, emergency procedure
Preoperative	Use of preoperative antibiotic prophylaxis (dose and agent), timing of preoperative antibiotic prophylaxis, use of mechanical bowel preparation, haemoglobin (g/dL), use of antibiotic bowel preparation, cytostatic chemotherapy, radiotherapy
Intraoperative	Surgical procedure(s), organ involvement, contamination (CDC wound classification(36)), laparoscopic surgery, mean arterial pressure (MAP) (mmHg), haemoglobin (g/dL), heart rate (beats/min), hemodynamic monitoring method, hemodynamic management algorithm, crystalloid infusion (ml), colloid Infusion (ml), red-cell transfusion (units), duration of surgery (min), duration of anaesthesia (min), mean core temperature (°C), lowest core temperature (°C), mean net fluid supplementation (ml/kg/hr), arterial oxygen saturation (%),

For peer review only - http://bmjopen.bmj.com/site/about/guidelines.xhtml

	peripheral oxygen saturation (%), subcutaneous oxygen tension (mm Hg), muscle oxygen tension (mm Hg), partial pressure of arterial oxygen (mmHg), mean FiO_2 (%), mean PEEP (cmH_2O), ventilator flow (L/min), peak airway pressure (mmHg), plateau pressure (mmHg), tidal Volume (ml/kg predicted body weight), respiratory frequency, vasopressor agent, vasopressor agent used (mg), glucose (mg/dL), use of general anaesthesia, use of spinal or epidural anaesthesia, use of mechanical ventilation, use of nitrous oxide, total blood loss (ml), fluids (ml), end tidal CO_2
Postoperative	Use of postoperative antibiotics (dose and agent), postoperative antibiotic duration (days), cytostatic chemotherapy, radiotherapy, haemoglobin (g/dL), VAS pain score, use of postoperative oxygen suppletion (duration, method and FiO_2), haemoglobin (g/dL), peripheral oxygen saturation (%), partial pressure of arterial oxygen (mmHg), subcutaneous oxygen tension (mmHg), muscle oxygen tension (mmHg), glucose (mg/dL), NNIS score(37), SSI risk score(38)
Oxygen Administration & Monitoring	Total duration and concentration of oxygen exposure during the pre/intra/postoperative period (timing of initiation, concentration, duration), oxygen supply and mode of administration (Intubation, use and type of face mask, nasal prongs), carrier gas (N_2, N_20, medical or room air), protocol-defined target or range of partial pressure of arterial oxygen (mmHg) or peripheral oxygen saturation (%)

Table 2. Outcome data and effect measure specification

Primary	- SSI within 90 days after surgery by the author's discretion
Secondary	- All-cause mortality within the longest available follow-up
Exploratory	- Survival within the longest available follow-up<br>- Serious adverse events defined by the ICH guidelines for good clinical practice(39)<br>- SSI monitored according to the CDC criteria and specified as either superficial, deep, organ/space(40)<br>- Respiratory insufficiency: defined as the need for respiratory assistance provided as ventilator therapy or non-invasive ventilation within 90 days after surgery<br>- Unplanned ICU admission (not part of routine postoperative care) (days)<br>- Hospital readmissions within 90 days after surgery<br>- Anastomotic leakage as defined by the international study group of rectal cancer(41)<br>- Total duration of hospitalization, including readmissions related to the initial hospitalization<br>- Any cardiovascular complication at any time after surgery<br>- Any pulmonary complications at any time after surgery<br>- Stroke at any time after surgery<br>- New or recurrent cancer diagnosis at any time after surgery<br>- Any further clinically relevant outcome reported in the IPD
* When patients are reoperated within follow up for reasons other than surgical site infection these cases will be excluded from the analysis based on loss to follow up.	

Missing data

For peer review only - http://bmjopen.bmj.com/site/about/guidelines.xhtml

When variables are missing at the participant level and the missing at random assumption is plausible, multiple imputations by chained equations may be applied in each trial separately before proceeding with the analysis. Variables that miss systematically i.e., unknown for the entire study, will not be imputated. When this concerns variables included in pre-defined analysis, studies systematically missing this variable will be excluded from that analysis. When this concerns variables not included in pre-defined analysis, these variables will be dropped from the main outcome analysis as potential confounding variables. The set of available variables for the main analysis will thus be determined by the data set with the least available variables. Variables from richer sets will remain available for exploratory analysis among data sets with the variable available.

Individual Participant Data integrity

We will check IPD for potential missing, invalid, or out-of-range values, inconsistencies, and discrepancies with the aggregate publication. When identified, we will seek to resolve the issues with the trial investigators to improve data quality and ensure that trials are represented accurately. In addition, any modelling assumptions made in the initial analysis will be evaluated (i.e., missing at random in the case of multiple imputation or non-informative censoring and proportional hazards in the case of time to event data). In the case of any concerns on IPD integrity further prove of execution of the trial and substantiation of the results may be requested such as prove of institutional review board approval or original case record forms. If concerns cannot be resolved with the trial investigators, the data of the concerning study will not be included in the primary analysis and the reason for exclusion will be explicitly stated.

Risk of bias

Two reviewers will independently assess the quality of the included studies using the Cochrane risk-of-bias tool for randomised trials (Rob 2).(42) Studies will be judged as "*low risk", "some concerns", or "high risk* of bias". Publication bias will be assessed using a contour enhanced funnel plot.(43) Additionally, the IPD will be used to directly check process parameters of some of the bias domains. Randomization and allocation concealment will be assessed by checking baseline imbalances. Incomplete outcome data will be assessed by checking the IPD to ensure all randomized patients are included. All available clinically relevant outcomes in the IPD will be reported in the IPD MA. For time to event outcomes such as mortality, pattern and extent of follow up will be checked. When needed,

For peer review only

additional follow up with original authors will be conducted to rectify any imbalances as far as possible.

Synthesis methods

All outcomes will be analysed according to the intention to treat principle and using a one-step approach for IPD MA. In the one-step approach, IPD will be modelled from all studies simultaneously using the generalised linear mixed model framework and the statistical model appropriate for the type of outcome being analysed (i.e., logistic regression for binary outcome data, linear regression for continuous outcome data, and Cox-regression for time to event data). A random treatment effect term will be added to the model and all other parameters (intercepts, prognostic factor effects and residual variances) will be stratified by trial to account for the clustering of patients within studies. Maximum likelihood with quadrature will be used as estimation method and study-specific centering of the variables.(44) Variables potentially affecting the outcome that, despite randomisation, show baseline imbalances across treatment arms will be considered for adjustment based on the criteria for confounder selection by VanderWeele and Shpitser.(45-48) Procedure duration is considered an important proxy for the complexity of the procedure and will also be considered for adjustment despite being measured during the exposure.(47, 48) We assume that the FiO_2 used does not affect procedure duration. Benjamini-Hochberg correction will be used to account for multiple testing for the primary and secondary outcomes when appropriate.(49)

Exploration of variation in effects

To explore the causes of heterogeneity and identify factors modifying the effects of high intraoperative FiO_2, we will perform pre-specified subgroup analyses by extending the one-step meta-analysis framework to include treatment-covariate interaction terms. Subgroups will be defined according to mean core temperature (<35°C), mean net fluid supplementation (<15ml/kg/hr), use of mechanical ventilation, use of nitrous oxide, use of preoperative antibiotic prophylaxis, and procedure duration (>2.5h). All subgroup variables have been proposed as effect modifiers in previous studies and have a plausible biological substantiation.(50-61) Cut-offs are driven by previously reported data.(50-61) Treatment-covariate interaction terms $p < 0.05$ will be considered statistically significant. Dose-response variation will be explored by total O_2 exposure duration for each primary outcome.

For peer review only - http://bmjopen.bmj.com/site/about/guidelines.xhtml

Additional analysis

A sensitivity analysis will be conducted to test the impact of excluding trials using N_2O as a carrier gas on the pooled effect estimate. In the case of exclusion of trials due to concerns on IPD integrity, a sensitivity analysis will be conducted to test the impact of including the concerning data on the pooled effect estimate. When multiple imputation is performed, a complete case analysis will also be conducted. In studies with sufficiently detailed data on the intervention, all analyses will also be conducted according to the per-protocol principle after adjustment for confounding factors due to incomplete adherence to the assigned treatments or use of off-protocol concomitant therapies according to the variable selection principles described for the primary analysis. Per protocol treatment will be defined as an FiO_2 of 0.80 ± 0.05 for at least 75% of the ventilation time in the intervention group, and an FiO_2 smaller than 0.40 with a margin of 0.05, for 75% in the control group. Patients requiring more oxygen for medical reasons, for example to maintain adequate saturation, after initial ventilation with an FiO_2 of 0.45 are exempted and not considered a protocol deviation. A sensitivity analysis will be conducted according to the two-step approach. All studies will be reanalysed separately, similarly to the one-step approach but without the term for trial clustering. The new aggregate data of each study will then be synthesised in a second step synthesising an overall estimate using maximum likelihood method followed by the Hartung-Knapp Sidik-Jonkman correction assuming random effects.(62) Between-study variance will be evaluated using τ^2; in addition, the Chi^2 test for heterogeneity will be performed with $p < 0.100$ considered statistically significant.. In the unlikely event that IPD will not be made available, aggregate study data will be included in the analyses during step two. Any unforeseen challenge during the analysis or choice that leads to discussion in the steering group that cannot be resolved by consensus will also be subjected to sensitivity analysis. For time to event outcomes a survival curve will be added to the Cox-regression analysis.

Certainty of the cumulative estimate

The Grading of Recommendations Assessment Development and Evaluation (GRADE) working group methodology will be used to assess the overall quality of evidence for the following domains: risk of bias, unexplained inconsistency, indirectness, imprecision, publication bias, and magnitude of effect. Additional domains may be considered where appropriate. Optimal information size, defined as the number of participants needed for a single adequately powered trial, was calculated assuming a type-1 error (α) of 0.05, a type 2 error (β) of 0.2 and a relative risk reduction of 0.25.(63) If a confidence interval failed to exclude appreciable benefit or harm, defined as a relative risk reduction

For peer review only - http://bmjopen.bmj.com/site/about/guidelines.xhtml

or increase of 0.25, the quality of evidence will be downgraded regardless of the optimal information size.(63) The overall certainty will be classified using four levels: high, moderate, low, and very low.(64)

Software

Results will be processed using R 4.0.4.

ETHICS AND DISSEMINATION

Ethical approval

Ethical approval is not deemed necessary for this study protocol.

Dissemination

This protocol and the results of this study will be submitted to a peer-reviewed medical journal regardless of the outcome. The protocol will be submitted before the data is gathered and analysed.

Competing interests

S.W.J. reports receipt of grants from Photonics in Healthcare, Integra LifeSciences, and Ethicon outside the submitted. P.S.M. reports receipt of grants or contracts from the Australian National Health and Medical Research Council (NHMRC), Practitioner Fellowship and Projects Grants, payment of expert testimony from Avant Medical Indemnity, and participation on a Data Safety Monitoring Board of Advisory Board for the SNAP, TOPIC-2 and BONANZA trials. A.F. reports receipt of institutional grants from the Australian Research Council Discovery Project and National Health and Medical Research Council Ideas outside the submitted work and participation on a Data Safety Monitoring Board or Advisory Board for the Australian Kidney Trials Research Network (INCH-HD, IMPEDE, TEQCH-PD, PHOSPHATE, BEST Fluids, N3RO trial, CKD-FIX, IMPROVE-FIX). R.G. reports participation on Steering Committee for the IntuBot Innosuisse Projekt and has a leadership role as ERC Director of Guidelines and ILCOR, and ILCOR Task Force Chair on Education, Implementation and Team, and Treasurer of European Airway Management society, and reports receipt without any payment of airway equipment for the research of the following: Intersurgical, Karl Storz, Verathon Inc, Aircraft Medical, Prodol Meditec, Venner Medical, Kingsystems, Medtronic, Ambu, VBM,

For peer review only - http://bmjopen.bmj.com/site/about/guidelines.xhtml

Radiometer, Sentec, and Fisher & Paykel. A.K. reports receipt of grants or contracts from Potrero Medical, Rehabtronics and The 37company outside the submitted work and participation on a Data Safety Monitoring Board of Advisory Board in Directedsystems, Potrero Medical and BioAgel Laboratories. J.M. reports voluntary participation as a panellist in the updated WHO Guidelines on high vs low FiO2 in 2018 and voluntary co-investigator in the PENGUIN trial of high versus low FiO2 for SSI prevention in abdominal surgery in low- and middle-income settings with GlobalSurg Collaborative. M.G.W.D. reports participation on a Data Safety Monitoring Board or Advisory Board for the following trials: DANCE, SPHINX, ICONIC, SAFE, PACER, LEARNS, RECAP, and BIOPEX2. C.F. reports receipt fees for lectures and educational events from Getinge and Medtronic outside the submitted work. C.S. reports receipts of institutional grants from NICHD outside the submitted work. M.A.B. reports receipt of institutional grants from KCI/3M, Johnson & Johnson, New Compliance, BD Bard, Gore, Telabio, GDM, Medtronic, and Smith & Nephew outside the submitted work, and participation on the Data Monitoring Committee of the EXTEND trial. M.W.H. reports receipt of institutional grants from ZonMw outside the submitted work, consulting institutional fees from IDD Pharma outside the submitted work, institutional payment or honoraria for lectures, presentations, speakers bureaus, manuscript writing or educational events from CSL Behring outside the submitted work, and has a leadership role in DGAI, ISAP and IARS (Anaesthesia and Analgesia). The other authors declare no conflict of interest.

Funding

This work was supported by the Amsterdams Universiteitsfonds grant (4069) to R. H. Hulskes.

Contributions

MH and MB are the guarantors of the study. SJ conceived the study. SJ and RH drafted the study protocol. All authors provided expertise from their respective area of interest, contributed to the study protocol, provided critical feedback, and approved the final manuscript. JM and BA provided independent advice on behalf of their respective organisations.

Acknowledgements

The authors thank Faridi Jamaludin as professional information specialist for the search.

For peer review only - http://bmjopen.bmj.com/site/about/guidelines.xhtml

368 **Data sharing**

369 Data will be made available upon reasonable request and review and approval of all collaborators.

For peer review only

For peer review only - http://bmjopen.bmj.com/site/about/guidelines.xhtml

References

1. Magill SS, Edwards JR, Bamberg W, Beldavs ZG, Dumyati G, Kainer MA, et al. Multistate point-prevalence survey of health care-associated infections. The New England journal of medicine. 2014;370(13):1198-208.
2. de Lissovoy G, Fraeman K, Hutchins V, Murphy D, Song D, Vaughn BB. Surgical site infection: incidence and impact on hospital utilization and treatment costs. American journal of infection control. 2009;37(5):387-97.
3. Control ECfDPa. Point prevalence survey of healthcareassociated infections and antimicrobial use in European acute care hospitals. Stockholm: ECDC; 2013.
4. ECDC. Surveillance of surgical site infections in Europe 2010–2011. Stockholm: ECDC; 2013.
5. Leaper DJ, van Goor H, Reilly J, Petrosillo N, Geiss HK, Torres AJ, et al. Surgical site infection - a European perspective of incidence and economic burden. Int Wound J. 2004;1(4):247-73.
6. Koek MBG, van der Kooi TII, Stigter FCA, de Boer PT, de Gier B, Hopmans TEM, et al. Burden of surgical site infections in the Netherlands: cost analyses and disability-adjusted life years. J Hosp Infect. 2019;103(3):293-302.
7. Allegranzi B, Bischoff P, de Jonge S, Kubilay NZ, Zayed B, Gomes SM, et al. New WHO recommendations on preoperative measures for surgical site infection prevention: an evidence-based global perspective. Lancet Infect Dis. 2016;16(12):e276-e87.
8. Allegranzi B, Zayed B, Bischoff P, Kubilay NZ, de Jonge S, de Vries F, et al. New WHO recommendations on intraoperative and postoperative measures for surgical site infection prevention: an evidence-based global perspective. Lancet Infect Dis. 2016;16(12):e288-e303.
9. Berrios-Torres SI, Umscheid CA, Bratzler DW, Leas B, Stone EC, Kelz RR, et al. Centers for Disease Control and Prevention Guideline for the Prevention of Surgical Site Infection, 2017. JAMA Surg. 2017.
10. Hedenstierna G, Perchiazzi G, Meyhoff CS, Larsson A. Who Can Make Sense of the WHO Guidelines to Prevent Surgical Site Infection? Anesthesiology. 2017;126(5):771-3.
11. Volk T, Peters J, Sessler DI. The WHO recommendation for 80% perioperative oxygen is poorly justified. Anaesthesist. 2017;66(4):227-9.
12. Wenk M, Van Aken H, Zarbock A. The New World Health Organization Recommendations on Perioperative Administration of Oxygen to Prevent Surgical Site Infections: A Dangerous Reductionist Approach? Anesth Analg. 2017;125(2):682-7.
13. Meyhoff CS, Fonnes S, Wetterslev J, Jorgensen LN, Rasmussen LS. WHO Guidelines to prevent surgical site infections. Lancet Infect Dis. 2017;17(3):261-2.
14. Mellin-Olsen J, McDougall RJ, Cheng D. WHO Guidelines to prevent surgical site infections. Lancet Infect Dis. 2017;17(3):260-1.
15. Berrios-Torres SI, Umscheid CA, Bratzler DW, Leas B, Stone EC, Kelz RR, et al. Centers for Disease Control and Prevention Guideline for the Prevention of Surgical Site Infection, 2017. JAMA Surg. 2017;152(8):784-91.
16. Solomkin J, Egger M, de Jonge S, Latif A, Loke YK, Berenholtz S, et al. World Health Organization Responds to Concerns about Surgical Site Infection Prevention Recommendations. Anesthesiology. 2018;128(1):221-2.
17. Akca O, Ball L, Belda FJ, Biro P, Cortegiani A, Eden A, et al. WHO Needs High FIO2? Turk J Anaesthesiol Reanim. 2017;45(4):181-92.
18. Weenink RP, de Jonge SW, Preckel B, Hollmann MW. PRO: Routine hyperoxygenation in adult surgical patients whose tracheas are intubated. Anaesthesia. 2020;75(10):1293-6.
19. Sperna Weiland NH, Berger MM, Helmerhorst HJF. CON: Routine hyperoxygenation in adult surgical patients whose tracheas are intubated. Anaesthesia. 2020;75(10):1297-300.
20. Weenink RP, de Jonge SW, van Hulst RA, Wingelaar TT, van Ooij PAM, Immink RV, et al. Perioperative Hyperoxyphobia: Justified or Not? Benefits and Harms of Hyperoxia during Surgery. J Clin Med. 2020;9(3).

For peer review only - http://bmjopen.bmj.com/site/about/guidelines.xhtml

21. Mattishent K, Thavarajah M, Sinha A, Peel A, Egger M, Solomkin J, et al. Safety of 80% vs 30-35% fraction of inspired oxygen in patients undergoing surgery: a systematic review and meta-analysis. Br J Anaesth. 2019;122(3):311-24.
22. de Jonge S, Egger M, Latif A, Loke YK, Berenholtz S, Boermeester M, et al. Effectiveness of 80% vs 30-35% fraction of inspired oxygen in patients undergoing surgery: an updated systematic review and meta-analysis. Br J Anaesth. 2019;122(3):325-34.
23. Global Guidelines for the Prevention of Surgical Site Infection, (2018).
24. Staehr-Rye AK, Meyhoff CS, Scheffenbichler FT, Vidal Melo MF, Gätke MR, Walsh JL, et al. High intraoperative inspiratory oxygen fraction and risk of major respiratory complications. BJA: British Journal of Anaesthesia. 2017;119(1):140-9.
25. Greif R, Akça O, Horn EP, Kurz A, Sessler DI. Supplemental perioperative oxygen to reduce the incidence of surgical-wound infection. New England journal of medicine. 2000;342(3):161-7.
26. Kurz A, Kopyeva T, Suliman I, Podolyak A, You J, Lewis B, et al. Supplemental oxygen and surgical-site infections: an alternating intervention controlled trial. Br J Anaesth. 2018;120(1):117-26.
27. Kurz A, Sessler DI, Lenhardt R. Perioperative normothermia to reduce the incidence of surgical-wound infection and shorten hospitalization. Study of Wound Infection and Temperature Group. The New England journal of medicine. 1996;334(19):1209-15.
28. Atkinson TM, Giraud GD, Togioka BM, Jones DB, Cigarroa JE. Cardiovascular and Ventilatory Consequences of Laparoscopic Surgery. Circulation. 2017;135(7):700-10.
29. Doherty M, Buggy DJ. Intraoperative fluids: how much is too much? Br J Anaesth. 2012;109(1):69-79.
30. Sessler DI. Perioperative thermoregulation and heat balance. Lancet. 2016;387(10038):2655-64.
31. Riley RD, Lambert PC, Abo-Zaid G. Meta-analysis of individual participant data: rationale, conduct, and reporting. BMJ. 2010;340:c221.
32. Stewart LA, Clarke M, Rovers M, Riley RD, Simmonds M, Stewart G, et al. Preferred Reporting Items for Systematic Review and Meta-Analyses of individual participant data: the PRISMA-IPD Statement. JAMA. 2015;313(16):1657-65.
33. Oxman AD, Clarke MJ, Stewart LA. From science to practice. Meta-analyses using individual patient data are needed. JAMA. 1995;274(10):845-6.
34. Stewart LA, Parmar MK. Meta-analysis of the literature or of individual patient data: is there a difference? Lancet. 1993;341(8842):418-22.
35. Shamseer L, Moher D, Clarke M, Ghersi D, Liberati A, Petticrew M, et al. Preferred reporting items for systematic review and meta-analysis protocols (PRISMA-P) 2015: elaboration and explanation. BMJ. 2015;350:g7647.
36. Garner JS. CDC guideline for prevention of surgical wound infections, 1985. Supersedes guideline for prevention of surgical wound infections published in 1982. (Originally published in November 1985). Revised. Infect Control. 1986;7(3):193-200.
37. Culver DH, Horan TC, Gaynes RP, Martone WJ, Jarvis WR, Emori TG, et al. Surgical wound infection rates by wound class, operative procedure, and patient risk index. National Nosocomial Infections Surveillance System. Am J Med. 1991;91(3B):152S-7S.
38. van Walraven C, Musselman R. The Surgical Site Infection Risk Score (SSIRS): A Model to Predict the Risk of Surgical Site Infections. PLoS One. 2013;8(6):e67167.
39. INTEGRATED ADDENDUM TO ICH E6(R1): GUIDELINE FOR GOOD CLINICAL PRACTICE E6(R2), (2016).
40. Mangram AJ, Horan TC, Pearson ML, Silver LC, Jarvis WR. Guideline for Prevention of Surgical Site Infection, 1999. Centers for Disease Control and Prevention (CDC) Hospital Infection Control Practices Advisory Committee. American journal of infection control. 1999;27:97-132.
41. Rahbari NN, Weitz J, Hohenberger W, Heald RJ, Moran B, Ulrich A, et al. Definition and grading of anastomotic leakage following anterior resection of the rectum: a proposal by the International Study Group of Rectal Cancer. Surgery. 2010;147(3):339-51.

For peer review only - http://bmjopen.bmj.com/site/about/guidelines.xhtml

42. Sterne JAC, Savovic J, Page MJ, Elbers RG, Blencowe NS, Boutron I, et al. RoB 2: a revised tool for assessing risk of bias in randomised trials. BMJ. 2019;366:l4898.

43. Peters JL, Sutton AJ, Jones DR, Abrams KR, Rushton L. Contour-enhanced meta-analysis funnel plots help distinguish publication bias from other causes of asymmetry. J Clin Epidemiol. 2008;61(10):991-6.

44. Riley RD, Legha A, Jackson D, Morris TP, Ensor J, Snell KIE, et al. One-stage individual participant data meta-analysis models for continuous and binary outcomes: Comparison of treatment coding options and estimation methods. Statistics in Medicine. 2020;39(19):2536-55.

45. VanderWeele TJ. Principles of confounder selection. Eur J Epidemiol. 2019;34(3):211-9.

46. VanderWeele TJ, Shpitser I. A new criterion for confounder selection. Biometrics. 2011;67(4):1406-13.

47. Haley RW, Culver DH, Morgan WM, White JW, Emori TG, Hooton TM. Identifying patients at high risk of surgical wound infection. A simple multivariate index of patient susceptibility and wound contamination. Am J Epidemiol. 1985;121(2):206-15.

48. Korol E, Johnston K, Waser N, Sifakis F, Jafri HS, Lo M, et al. A systematic review of risk factors associated with surgical site infections among surgical patients. PLoS One. 2013;8(12):e83743.

49. Thissen D, Steinberg L, Kuang D. Quick and Easy Implementation of the Benjamini-Hochberg Procedure for Controlling the False Positive Rate in Multiple Comparisons. Journal of Educational and Behavioral Statistics. 2002;27(1):77-83.

50. Pryor KO, Fahey TJ, 3rd, Lien CA, Goldstein PA. Surgical site infection and the routine use of perioperative hyperoxia in a general surgical population: a randomized controlled trial. JAMA. 2004;291(1):79-87.

51. Belda FJ, Aguilera L, Garcia de la Asuncion J, Alberti J, Vicente R, Ferrandiz L, et al. Supplemental perioperative oxygen and the risk of surgical wound infection: a randomized controlled trial. JAMA. 2005;294(16):2035-42.

52. Meyhoff CS, Wetterslev J, Jorgensen LN, Henneberg SW, Hogdall C, Lundvall L, et al. Effect of high perioperative oxygen fraction on surgical site infection and pulmonary complications after abdominal surgery: the PROXI randomized clinical trial. JAMA. 2009;302(14):1543-50.

53. Bickel A, Gurevits M, Vamos R, Ivry S, Eitan A. Perioperative hyperoxygenation and wound site infection following surgery for acute appendicitis: a randomized, prospective, controlled trial. Arch Surg. 2011;146(4):464-70.

54. Duggal N, Poddatoori V, Noroozkhani S, Siddik-Ahmad RI, Caughey AB. Perioperative oxygen supplementation and surgical site infection after cesarean delivery: a randomized trial. Obstet Gynecol. 2013;122(1):79-84.

55. Gardella C, Goltra LB, Laschansky E, Drolette L, Magaret A, Chadwick HS, et al. High-concentration supplemental perioperative oxygen to reduce the incidence of postcesarean surgical site infection: a randomized controlled trial. Obstet Gynecol. 2008;112(3):545-52.

56. Mayzler O, Weksler N, Domchik S, Klein M, Mizrahi S, Gurman GM. Does supplemental perioperative oxygen administration reduce the incidence of wound infection in elective colorectal surgery? Minerva Anestesiol. 2005;71(1-2):21-5.

57. Myles PS, Leslie K, Chan MT, Forbes A, Paech MJ, Peyton P, et al. Avoidance of nitrous oxide for patients undergoing major surgery: a randomized controlled trial. Anesthesiology. 2007;107(2):221-31.

58. Stall A, Paryavi E, Gupta R, Zadnik M, Hui E, O'Toole RV. Perioperative supplemental oxygen to reduce surgical site infection after open fixation of high-risk fractures: a randomized controlled pilot trial. J Trauma Acute Care Surg. 2013;75(4):657-63.

59. Scifres CM, Leighton BL, Fogertey PJ, Macones GA, Stamilio DM. Supplemental oxygen for the prevention of postcesarean infectious morbidity: a randomized controlled trial. Am J Obstet Gynecol. 2011;205(3):267 e1-9.

60. Williams NL, Glover MM, Crisp C, Acton AL, McKenna DS. Randomized controlled trial of the effect of 30% versus 80% fraction of inspired oxygen on cesarean delivery surgical site infection. Am J Perinatol. 2013;30(9):781-6.

For peer review only - http://bmjopen.bmj.com/site/about/guidelines.xhtml

61. Thibon P, Borgey F, Boutreux S, Hanouz JL, Le Coutour X, Parienti JJ. Effect of perioperative oxygen supplementation on 30-day surgical site infection rate in abdominal, gynecologic, and breast surgery: the ISO2 randomized controlled trial. Anesthesiology. 2012;117(3):504-11.

62. Langan D, Higgins JPT, Jackson D, Bowden J, Veroniki AA, Kontopantelis E, et al. A comparison of heterogeneity variance estimators in simulated random-effects meta-analyses. Res Synth Methods. 2019;10(1):83-98.

63. Schünemann H, Brożek J, Guyatt G, Oxman A. Handbook for grading the quality of evidence and the strength of recommendations using the GRADE approach (updated October 2013). GRADE Working Group, 2013.

64. Guyatt GH, Oxman AD, Vist GE, Kunz R, Falck-Ytter Y, Alonso-Coello P, et al. GRADE: an emerging consensus on rating quality of evidence and strength of recommendations. BMJ. 2008;336(7650):924-6.

For peer review only

For peer review only - http://bmjopen.bmj.com/site/about/guidelines.xhtml

PRISMA-P (Preferred Reporting Items for Systematic review and Meta-Analysis Protocols) 2015 checklist: recommended items to address in a systematic review protocol*

Section and topic	Item No	Checklist item	Reported on page
**ADMINISTRATIVE INFORMATION**			
Title:			
Identification	1a	Identify the report as a protocol of a systematic review	1
Update	1b	If the protocol is for an update of a previous systematic review, identify as such	NA
Registration	2	If registered, provide the name of the registry (such as PROSPERO) and registration number	2
Authors:			
Contact	3a	Provide name, institutional affiliation, e-mail address of all protocol authors; provide physical mailing address of corresponding author	1
Contributions	3b	Describe contributions of protocol authors and identify the guarantor of the review	13
Amendments	4	If the protocol represents an amendment of a previously completed or published protocol, identify as such and list changes; otherwise, state plan for documenting important protocol amendments	6
Support:			
Sources	5a	Indicate sources of financial or other support for the review	13
Sponsor	5b	Provide name for the review funder and/or sponsor	13
Role of sponsor or funder	5c	Describe roles of funder(s), sponsor(s), and/or institution(s), if any, in developing the protocol	13
**INTRODUCTION**			
Rationale	6	Describe the rationale for the review in the context of what is already known	4-5
Objectives	7	Provide an explicit statement of the question(s) the review will address with reference to participants, interventions, comparators, and outcomes (PICO)	4
**METHODS**			
Eligibility criteria	8	Specify the study characteristics (such as PICO, study design, setting, time frame) and report characteristics (such as years considered, language, publication status) to be used as criteria for eligibility for the review	7
Information sources	9	Describe all intended information sources (such as electronic databases, contact with study authors, trial registers or other grey literature sources) with planned dates of coverage	7
Search strategy	10	Present draft of search strategy to be used for at least one electronic database, including planned limits, such that it could be repeated	Supplement01

For peer review only

Study records:			
Data management	11a	Describe the mechanism(s) that will be used to manage records and data throughout the review	8
Selection process	11b	State the process that will be used for selecting studies (such as two independent reviewers) through each phase of the review (that is, screening, eligibility and inclusion in meta-analysis)	7
Data collection process	11c any	Describe planned method of extracting data from reports (such as piloting forms, done independently, in duplicate), processes for obtaining and confirming data from investigators	8
Data items	12	List and define all variables for which data will be sought (such as PICO items, funding sources), any pre-planned data assumptions and simplifications	8-9
Outcomes and prioritization	13	List and define all outcomes for which data will be sought, including prioritization of main and additional outcomes, with rationale	9
Risk of bias in individual studies	14	Describe anticipated methods for assessing risk of bias of individual studies, including whether this will be done at the outcome or study level, or both; state how this information will be used in data synthesis	9-10
Data synthesis	15a	Describe criteria under which study data will be quantitatively synthesised	11
	15b	If data are appropriate for quantitative synthesis, describe planned summary measures, methods of handling data and methods of combining data from studies, including any planned exploration of consistency (such as I^2, Kendall's τ)	12
	15c	Describe any proposed additional analyses (such as sensitivity or subgroup analyses, meta-regression)	12
	15d	If quantitative synthesis is not appropriate, describe the type of summary planned	NA
Meta-bias(es)	16	Specify any planned assessment of meta-bias(es) (such as publication bias across studies, selective reporting within studies)	10
Confidence in cumulative evidence	17	Describe how the strength of the body of evidence will be assessed (such as GRADE)	12-13

*** It is strongly recommended that this checklist be read in conjunction with the PRISMA-P Explanation and Elaboration (cite when available) for important clarification on the items. Amendments to a review protocol should be tracked and dated. The copyright for PRISMA-P (including checklist) is held by the PRISMA-P Group and is distributed under a Creative Commons Attribution Licence 4.0.**

From: Shamseer L, Moher D, Clarke M, Ghersi D, Liberati A, Petticrew M, Shekelle P, Stewart L, PRISMA-P Group. Preferred reporting items for systematic review and meta-analysis protocols (PRISMA-P) 2015: elaboration and explanation. BMJ. 2015 Jan 2;349(jan02 1):g7647.

For peer review only

BMJ Open

The benefits and harms of perioperative high fraction inspired oxygen for surgical site infection prevention: a protocol for a systematic review and meta-analysis of individual patient data of randomised controlled trials

Journal:	*BMJ Open*
Manuscript ID	bmjopen-2022-067243.R1
Article Type:	Protocol
Date Submitted by the Author:	11-Apr-2023
Complete List of Authors:	de Jonge, Stijn; Amsterdam UMC Locatie AMC, Surgery; Amsterdam UMC Locatie AMC, Anaesthesiology<br>Hulskes, Rick; Amsterdam UMC Locatie AMC, Anaesthesiology; Amsterdam UMC Locatie AMC, Surgery<br>Zokaei Nikoo, Maedeh; Cleveland Clinic Foundation, Outcomes research<br>Weenink, Robert ; Amsterdam UMC Locatie AMC, Anaesthesiology<br>Meyhoff, Christian; Copenhagen University Hospital, Anaesthesia and Intensive Care<br>Leslie, Kate; The Royal Melbourne Hospital, Anaesthesia<br>Myles, Paul; Monash University, Anaesthesia and Perioperative Medicine<br>Forbes, Andrew; Monash University, Epidemiology and Preventive Medicine<br>Greif, Robert ; Inselspital Universitatsspital Bern, Anaesthesiology and PAin Therapy<br>Akca, Ozan; Johns Hopkins University, Anaesthesiology & Critical Care Medicine<br>Kurz, Andrea; Cleveland Clinic, General Anesthesiology<br>Sessler, Daniel; Cleveland Clinic,<br>Martin, Janet; University of Western Ontario, Anaesthesiology & Perioperative Medicine and Department of Epidemiology & Biostatistics<br>Dijkgraaf, Marcel; Amsterdam UMC Locatie AMC, Epidemiology and Data Science<br>Pryor, Kane; Weill Cornell Medical College, Anesthesiology<br>Belda, F. Javier; Hospital Clinico Universitario, Surgery; Hospital Clinico Universitario, Anaesthesia and Critical Care<br>Ferrando, Carlos; Hospital Clinico Universitario, Anesthesiology and Cirtical Care<br>Gurman, Gabriel; Ben-Gurion University of the Negev, Anaesthesiology and Critical Care Medicine<br>Scifres, Christina; Indiana University School of Medicine,<br>McKenna, David; Wright State University, Obstetrics and Gynaecology<br>Chan, MTV; The Chinese University of Hong Kong, Anesthesia and Intensive Care<br>Thibon, Pascal; Centre d'appui pour la prevention des infections associees aux soins d'Ile-de-France<br>Mellin-Olsen, Jannicke; World Federation of Societies of Anesthesiologists<br>Allegranzi, B; World Health Organization<br>Boermeester, Marja; Amsterdam UMC Locatie AMC, Surgery

	Hollmann, Markus; Amsterdam UMC Locatie AMC, Anaesthesiology
**Primary Subject Heading**:	Anaesthesia
Secondary Subject Heading:	Surgery
Keywords:	ANAESTHETICS, WOUND MANAGEMENT, Infection control < INFECTIOUS DISEASES, SURGERY

SCHOLARONE™ Manuscripts

TITLE PAGE

The benefits and harms of perioperative high fraction inspired oxygen for surgical site infection prevention: a protocol for a systematic review and meta-analysis of individual patient data of randomised controlled trials

S. W. de Jonge[1a,b,2*], R. H. Hulskes[1a,2], M. Zokaei Nikoo[11], R. P. Weenink[2], C. S. Meyhoff[3], K. Leslie[4], P. S. Myles[5], A. Forbes[6], R. Greif[7,8], O. Akça[9], A. Kurz[10], D. I. Sessler[11], J. Martin[12], M. G. Dijkgraaf[13a,b], K. O. Pryor[14], F. J. Belda[15], C. Ferrando[16a,16b], G. M. Gurman[17], C. Scifres[18], D. S. McKenna[19], M. T. V. Chan[20], P. Thibon[21], J. Mellin-Olsen[22], B. A. Allegranzi[23], M. A. Boermeester[1a, b], M. W. Hollmann[2]

[1a] Department of Surgery, Amsterdam UMC, University of Amsterdam, Amsterdam, The Netherlands
[1b] Gastroenterology Endocrinology & Metabolism, Amsterdam, the Netherlands, Amsterdam, The Netherlands
[2] Department of Anaesthesiology, Amsterdam UMC, University of Amsterdam, Amsterdam, The Netherlands
[3] Department of Anaesthesia and Intensive Care, Copenhagen University Hospital – Bispebjerg and Frederiksberg, Copenhagen, Denmark
[4] Department of Critical Care, Melbourne Medical School, University of Melbourne, Australia
[5] Department of Anaesthesiology and Perioperative Medicine, Alfred Hospital and Monash University, Melbourne, Australia
[6] Department of Epidemiology and Preventive Medicine, Monash University, Melbourne, Australia
[7] Department of Anaesthesiology and Pain Medicine, Bern University Hospital, University of Bern, Bern, Switzerland
[8] School of Medicine, Sigmund Freud University Vienna, Vienna, Austria
[9] Department of Anaesthesiology & Critical Care Medicine, Johns Hopkins University, Maryland, United States of America
[10] Department of Anaesthesiology and department of Outcomes Research, Cleveland Clinic Cleveland Ohio, Ohio, United States of America
[11] Department of Outcomes Research, Cleveland Clinic Cleveland Ohio, Ohio, United States of America
[12] Department of Anaesthesiology & Perioperative Medicine and Department of Epidemiology & Biostatistics, University of Western Ontario, Canada
[13a] Amsterdam UMC location University of Amsterdam, Epidemiology and Data Science, Amsterdam, The Netherlands
[13b] Amsterdam Public Health, Methodology, Amsterdam, The Netherlands
[14] Department of Anaesthesiology, Weil Medical College of Cornell University, New York City, New York, United States of America
[15] Department of Surgery and Department of Anaesthesia Critical Care, Hospital Clinico Universitario De Valencia, Valencia, Spain
[16a] Department of Anaesthesiology and Critical Care, Hospital Clínic I Provincial, Barcelona, Spain
[16b] CIBER de Enfermedades Respiratorias, Instituto de Salud Carlos III, Madrid, Spain
[17] Department of Anaesthesiology and Critical Care Medicine, Ben Gurion University of the Negev, Beer Sheva, Israel
[18] Department of Obstetrics and Gynaecology, Indiana University School of Medicine, Indianapolis, United States of America
[19] Department of Obstetrics and Gynaecology, Wright State University and Miami Valley Hospital, Ohio, United States of America
[20] Department of Anaesthesia and Intensive Care, Chinese University of Hong Kong, Hong Kong Special Administrative Region, China
[21] Centre d'appui pour la Prévention des Infections Associées aux Soins, CPias Normandie, Centre Hospitalo-Universitaire, Caen, Normandy, France
[22] World Federation of Societies of Anaesthesiologists
[23] World Health Organization, Service Delivery and Safety, Infection Prevention and Control Global Unit

***Corresponding author**

Corresponding author: S. W. de Jonge, Amsterdam UMC, University of Amsterdam, Meibergdreef 9, 1105 AZ Amsterdam, the Netherlands, email: s.w.dejonge@amsterdamumc.nl, telephone number 0031 (0)20 566 9111.

Word count: 2921 (excluding title page, abstract, article summary, tables, ethics and dissemination, and references)

For peer review only - http://bmjopen.bmj.com/site/about/guidelines.xhtml

Keywords: general anaesthesia; hyperoxia; postoperative outcome; surgical site infection; surgical wound infection

For peer review only

For peer review only - http://bmjopen.bmj.com/site/about/guidelines.xhtml

ABSTRACT

Introduction: The use of high fraction of inspired oxygen (FiO_2) intraoperatively for the prevention of surgical site infection (SSI) remains controversial. Promising results of early randomized controlled trials (RCT) have been replicated with varying success and subsequent meta-analysis are equivocal. Recent advancements in perioperative care, including the increased use of laparoscopic surgery and pneumoperitoneum and shifts in fluid and temperature management, can affect peripheral oxygen delivery and may explain the inconsistency in reproducibility. However, the published data provides insufficient detail on the participant level to test these hypotheses. The purpose of this individual participant data meta-analysis is to assess the described benefits and harms of intraoperative high FiO_2 compared to regular (0.21-0.40) FiO_2 and its potential effect modifiers.

Methods and analysis:

Two reviewers will search medical databases and online trial registries, including MEDLINE, EMBASE, CENTRAL, CINHAL, and clinicaltrial.gov, for randomised and quasi randomised controlled trials comparing the effect of intraoperative high FiO2 (0.60-1.00) to regular FiO2 (0.21-0.40)on SSI within 90 days after surgery in adult patients. Secondary outcome will be all-cause mortality within the longest available follow-up. Investigators of the identified trials will be invited to collaborate. Data will be analysed with the one step approach using the generalised linear mixed model framework and the statistical model appropriate for the type of outcome being analysed (logistic and cox regression respectively), with a random treatment effect term to account for the clustering of patients within studies. The certainty of evidence will be assessed using GRADE methodology. Pre-specified subgroup analyses include use of mechanical ventilation, nitrous oxide, preoperative antibiotic prophylaxis, temperature (<35°C), fluid supplementation (<15ml/kg/hr) and procedure duration (>2.5h).

Ethics and dissemination: Ethics approval is not required. Investigators will de-identify individual participant data before it is shared. The results will be submitted to a peer-review journal.

Trial Registration Number: PROSPERO CRD42018090261

For peer review only - http://bmjopen.bmj.com/site/about/guidelines.xhtml

ARTICLE SUMMARY

Strengths and limitations of this study

- Individual participant data meta-analysis (IPD MA) of (quasi-)randomised controlled trials provides the best possible analysis of the available data on the participant level, permitting the investigation of potential effect modifiers.
- IPD MA requires the collaboration of all investigators that have published data on the relevant topic and leads to a broad consensus on the outcome and interpretation of the analysis
- IPD MA depends on the quality of data that is made available by the authors of the original studies.

For peer review only

For peer review only - http://bmjopen.bmj.com/site/about/guidelines.xhtml

INTRODUCTION

Surgical site infection (SSI) is one of the most common healthcare-associated infections and leads to morbidity, mortality, and longer hospital stay.(1-4) The attributable costs can be more than €14.000 per SSI, and European totals are estimated to range from €1.5 to €19 billion per year.(5, 6) In 2016-2017, both the World Health Organization (WHO) and the Centres of Disease Control and Prevention (CDC) independently released evidence-based guidelines on the prevention of SSI that included a recommendation in favour of the administration of high fraction of inspired (FiO_2) for patients undergoing surgery under general anaesthesia.(7-9) This has led to a debate between opponents and proponents of the use of high FiO_2 in several editorials and correspondences across medical speciality literature.(10-20) Concerns were raised on the safety of the use of high FiO_2 as well as on the conflicting study results with some in support of the use of high FiO_2 to reduce SSI and some not.(10-20) Finally, studies by of one of the authors that contributed to the body of evidence were retracted because of unreproducible statistics.

In response to these concerns, the WHO conducted an independent systematic review on the safety of high intraoperative FiO_2 and updated the systematic review on its effectiveness, excluding the disputed trials.(21, 22) No evidence of harm to discourage the use of high FiO_2 was found, yet the evidence of an effect of SSI had become weaker, and the recommendation was adjusted accordingly.(23) Despite various studies and recommendations, there is still no consensus on the safety and effectiveness of using high FiO_2 during surgery with regard to SSI, all-cause mortality and other adverse events in adult patients. This leads to practice variation that inevitably exposes patients to suboptimal care.(24) There is a need for better understanding and consensus on this issue.

Since the early promising results, perioperative care has changed considerably. Open abdominal surgery has been largely replaced by laparoscopic surgery, fluid management has moved from liberal to restrictive, to advanced goal directed regimens and active perioperative warming has become a mainstay.(25-27) All these changes have considerable consequences for hemodynamic, microcirculation, and eventually peripheral oxygen delivery.(28-30) These changes may explain the inconsistency in reproducibility, but the available data provides insufficient detail on the participant level to test the potential of high FiO_2. Meta-analysis of individual participant data uses the raw individual-level data from the original study for synthesis and overcomes this limitation.(31, 32) IPD MA enables analysis of uniform outcomes with more statistical power and assessment of potential effect modifiers.(33, 34) Importantly, IPD MA requires collaboration with all published researchers on the topic leading to a broad consensus on the outcome of data analysis and interpretation.

For peer review only - http://bmjopen.bmj.com/site/about/guidelines.xhtml

The purpose of this IPD MA is to assess the potential benefits and harms of intraoperative high (0.60-1.00) FiO_2 compared to traditional (0.21-0.40) FiO_2 and its effect modifiers in adult patients undergoing surgery with SSI being the primary outcome. This IPD MA is initiated by the University of Amsterdam / Amsterdam University Medical Center, and encouraged by the WHO and the World Federation of Societies of Anaesthesiologists (WFSA) to provide patients and practitioners with the best possible evidence and guidance on this disputed area and will give clearance of the disputed hypothesis that high FiO_2 reduces the incidence of SSI.

For peer review only

For peer review only - http://bmjopen.bmj.com/site/about/guidelines.xhtml

METHODS AND ANALYSIS

Protocol and registration

This study protocol is registered with the International Prospective Register of Systematic Reviews (PROSPERO) on 7 March 2018 and was last updated on 15 July 2022 (registration number CRD42018090261). The study protocol is designed and written to adhere to the Preferred Reporting Items for Systematic Review and Meta-Analysis Protocols (PRISMA-P)(35) and the Preferred Reporting Items for Systematic Review Meta-Analysis of individual patient data (IPD) (PRISMA-IPD).(32)

Patient and Public Involvement Statement

This project is encouraged by the World Health Organization (WHO), and the World Federation of Societies of Anesthesiologists (WFSA) to provide patients and practitioners with the best possible evidence and guidance on this disputed area. WHO and WFSA have provided external independent review and advice on research direction and aim.

Governance

This study is an initiative of the Amsterdam University Medical Centre, encouraged by the WHO and the WFSA. Both organisations recognise the urgent need for this research and provide external independent review and advice. The writing committee consists of the study coordinator, two reviewers, a lead methodologist, and a principal investigator from both the surgery and the anaesthesiology department of the Amsterdam University Medical Centre and two external content matter experts. The writing committee is entirely independent of the initial trials and has full responsibility for all methodological decisions. A broader steering committee with representatives of the collaborating trial groups identified during the project will be invited to comment on and co-author the final protocol and IPD MA report. By sharing their IPD, collaborators will obtain one co-authorship on the IPD MA report and one additional co-authorship if data of more than 300 participants is shared. For transparency and against intellectual bias, a record will be kept of all comments. Any important amendments to the protocol will be recorded in PROSPERO record and discussed in the methods section of the final report.

Eligibility criteria

For peer review only - http://bmjopen.bmj.com/site/about/guidelines.xhtml
For peer review only

We will include all randomised and quasi-randomised controlled trials comparing the effect of intraoperative high FiO_2 (0.60-1.0) to traditional FiO_2 (0.21-0.40) in patients undergoing surgery. Definitions for high and low FiO_2 were determined by literature review and consensus among the IPDMA collaborators.(21, 22) These trials may include patients of any age undergoing surgery except for neonates, regardless publication, language, or year of conduct and should include at least data on age, sex, mean FiO_2 administered, method of oxygen administration, SSI, mortality, or other serious adverse events. Any outcome found to be recorded in these trials will be included in the analysis. Studies without random or quasi-random treatment allocation, animal studies, and studies outside of the intraoperative period will be excluded.

Identifying studies – information sources

The initial search conducted for the WHO guideline will be updated by a professional information specialist.(21, 22) Medical databases will be searched, including MEDLINE, EMBASE, CENTRAL, CINHAL, and the WHO regional databases. Online trial registries will be searched to identify potential unpublished evidence or any ongoing trials. The search will not be limited by language or date of publication. A final update will be conducted before the final round of revisions preceding submission for publication. The reference list of all included studies will be hand searched for any additional relevant trials not already identified through database searching. All corresponding authors of relevant clinical trials will be contacted to review the list of identified studies for the omission of potentially relevant studies missed by the search.

For peer review only

Study selection process

Two reviewers will independently assess articles retrieved by the search against the eligibility criteria. After screening the title and abstract, the full text of potentially eligible papers will be retrieved and assessed. When no full paper exists, or trial eligibility is in doubt, the study authors will be contacted to provide further information. Any discrepancies in study selection will be resolved through consensus and discussion with a senior author. All studies that pass title and abstract screening but were not eligible for inclusion will be listed with the reasons for exclusion.

Study collaboration invitation

Authors of eligible studies will be contacted and invited to collaborate on the IPD MA. An email invitation will be sent to the corresponding authors outlining the IPMDA goals. If no reply is received

For peer review only - http://bmjopen.bmj.com/site/about/guidelines.xhtml

within two weeks, a second email request will be sent to the corresponding and first author. If no response is received again, we will try to contact all authors by email and telephone. IPD data will be considered unavailable if numerous times (at least five) no reply is received if authors no longer have access to the study data or consent to collaboration.

Data collection process

The collaborating investigators will be requested to sign a data transfer agreement describing the ownership and storage of the IPD before IPD is shared. Whenever possible, data collection, interview on the protocol, and formal handoff on the data codebook will be done electronically via email, videoconference, or a suitable alternative. Whenever requested by the original investigator, a researcher will visit the investigators for a physical data transfer, in-person interview, and data codebook handoff. IPD will be de-identified by the suppling collaborator. The IPD de-identification code will not be shared. IPD will be transferred using one of the following secure methods: SurfFilesender, a secure password protected data transfer service,(36) end-to-end encrypted and password protected using email or send by courier on a physical storage media. Once transferred, IPD will be stored securely on the local server of the Amsterdam UMC where appropriate data and privacy policies will be maintained, as well as procedures and associated physical, technical and administrative safeguards to assure that the IPD are accessed only by authorized personnel. In the unlikely event that individual patient data will not be made available, the reason will be recorded. The aggregate data of the study will be used in a sensitivity analysis. Aggregate data collection will be performed as appropriate for a regular meta-analysis by two independent reviewers according to a predefined data extraction sheet and overseen by a senior author to settle potential discrepancies. The University of Amsterdam's Clinical Research Unit will facilitate secure data storage.

For peer review only

Data items

Data items will include all data recorded by the initial trial investigators including, but not limited to the items listed in table 1 and table 2. SSI within 90 days after surgery according to the authors discretion will be the primary outcome, all-cause mortality within the longest available follow up will be the secondary outcome. All other outcomes are exploratory.

**Table 1. Baseline and procedure characteristics**	
Baseline	Sex, age, BMI (kg/m^2), ASA physical status score, smoking status, peripheral vascular disease, diabetes, (metastatic) cancer, congestive heart disease, (pulmonary) hypertension, chronic obstructive pulmonary disease,

	immunosuppressant use, peripheral oxygen saturation (%), glucose (mg/dL), indication for surgery, emergency procedure
Preoperative	Use of preoperative antibiotic prophylaxis (dose and agent), timing of preoperative antibiotic prophylaxis, use of mechanical bowel preparation, haemoglobin (g/dL), use of antibiotic bowel preparation, cytostatic chemotherapy, radiotherapy, Use of preoperative skin preparation prophylaxis (dose and agent), Timing of preoperative skin preparation prophylaxis.
Intraoperative	Surgical procedure(s), organ involvement, contamination (CDC wound classification(37)), laparoscopic surgery, mean arterial pressure (MAP) (mmHg), haemoglobin (g/dL), heart rate (beats/min), hemodynamic monitoring method, hemodynamic management algorithm, crystalloid infusion (ml), colloid Infusion (ml), red-cell transfusion (units), duration of surgery (min), duration of anaesthesia (min), mean core temperature (°C)*, lowest core temperature (°C)*, mean net fluid supplementation (ml/kg/hr), arterial oxygen saturation (%), peripheral oxygen saturation (%), subcutaneous oxygen tension (mm Hg), muscle oxygen tension (mm Hg), partial pressure of arterial oxygen (mmHg), mean FiO_2 (%), mean PEEP (cmH_2O), ventilator flow (L/min), peak airway pressure (mmHg), plateau pressure (mmHg), tidal Volume (ml/kg predicted body weight), respiratory frequency, vasopressor agent, vasopressor agent used (mg), glucose (mg/dL), use of general anaesthesia, use of spinal or epidural anaesthesia, use of mechanical ventilation, use of nitrous oxide, total blood loss (ml), fluids (ml), end tidal CO_2
Postoperative	Use of postoperative antibiotics (dose and agent), postoperative antibiotic duration (days), cytostatic chemotherapy, radiotherapy, haemoglobin (g/dL), VAS pain score, use of postoperative oxygen suppletion (duration, method and FiO_2), haemoglobin (g/dL), peripheral oxygen saturation (%), partial pressure of arterial oxygen (mmHg), subcutaneous oxygen tension (mmHg), muscle oxygen tension (mmHg), glucose (mg/dL), NNIS score(38), SSI risk score(39), Use of drains
Oxygen Administration & Monitoring	Total duration and concentration of oxygen exposure during the pre/intra/postoperative period (timing of initiation, concentration, duration), oxygen supply and mode of administration (Intubation, use and type of face mask, nasal prongs), carrier gas (N_2, N_20, medical or room air), protocol-defined target or range of partial pressure of arterial oxygen (mmHg) or peripheral oxygen saturation (%)

* Direct measurement or its approximation by peripheral measurement

Table 2. Outcome data and effect measure specification

Primary	- SSI within 90 days after surgery by the author's discretion
Secondary	- All-cause mortality within the longest available follow-up
Exploratory	- Survival within the longest available follow-up<br>- Serious adverse events defined by the ICH guidelines for good clinical practice(40)<br>- SSI monitored according to the CDC criteria and specified as either superficial, deep, organ/space(41)<br>- Respiratory insufficiency: defined as the need for respiratory assistance provided as ventilator therapy or non-invasive ventilation within 90 days after surgery<br>- Unplanned ICU admission (not part of routine postoperative care) (days)

For peer review only - http://bmjopen.bmj.com/site/about/guidelines.xhtml

	- Hospital readmissions within 90 days after surgery<br>- Anastomotic leakage as defined by the international study group of rectal cancer(42)<br>- Total duration of hospitalization, including readmissions related to the initial hospitalization<br>- Any cardiovascular complication at any time after surgery<br>- Any pulmonary complications at any time after surgery<br>- Stroke at any time after surgery<br>- New or recurrent cancer diagnosis at any time after surgery<br>- Any further clinically relevant outcome reported in the IPD
* When patients are reoperated within follow up for reasons other than surgical site infection these cases will be excluded from the analysis based on loss to follow up.	

Missing data

When variables are missing at the participant level and the missing at random assumption is plausible, multiple imputations by chained equations may be applied in each trial separately before proceeding with the analysis. Variables that miss systematically i.e., unknown for the entire study or are deemed missing non-randomly after discussion in the writing committee, will not be imputated. When this concerns variables included in pre-defined analysis, studies systematically missing this variable will be excluded from that analysis. When this concerns variables not included in pre-defined analysis, these variables will be dropped from the main outcome analysis as potential confounding variables. The set of available variables for the main analysis will thus be determined by the data set with the least available variables. Variables from richer sets will remain available for exploratory analysis among data sets with the variable available.

Individual Participant Data integrity

We will check IPD for potential missing, invalid, or out-of-range values, inconsistencies, and discrepancies with the aggregate publication. When identified, we will seek to resolve the issues with the trial investigators to improve data quality and ensure that trials are represented accurately. In addition, any modelling assumptions made in the initial analysis will be evaluated (i.e., missing at random in the case of multiple imputation or non-informative censoring and proportional hazards in the case of time to event data). In the case of any concerns on IPD integrity further prove of execution of the trial and substantiation of the results may be requested such as prove of institutional review board approval or original case record forms. If concerns cannot be resolved with the trial investigators, the data of the concerning study will not be included in the primary analysis and the reason for exclusion will be explicitly stated.

For peer review only

For peer review only - http://bmjopen.bmj.com/site/about/guidelines.xhtml

Risk of bias

Two reviewers will independently assess the quality of the included studies using the Cochrane risk-of-bias tool for randomised trials (Rob 2).(43) Studies will be judged as "*low risk", "some concerns", or "high risk* of bias". Publication bias will be assessed using a contour enhanced funnel plot.(44) Additionally, the IPD will be used to directly check process parameters of some of the bias domains. Randomization and allocation concealment will be assessed by checking baseline imbalances. Incomplete outcome data will be assessed by checking the IPD to ensure all randomized patients are included. All available clinically relevant outcomes in the IPD will be reported in the IPD MA. For time to event outcomes such as mortality, pattern and extent of follow up will be checked. When needed, additional follow up with original authors will be conducted to rectify any imbalances as far as possible.

Synthesis methods

All outcomes will be analysed according to the intention to treat principle and using a one-step approach for IPD MA. In the one-step approach, IPD will be modelled from all studies simultaneously using the generalised linear mixed model framework and the statistical model appropriate for the type of outcome being analysed (i.e., logistic regression for binary outcome data, linear regression for continuous outcome data, and Cox-regression for time to event data). A random treatment effect term will be added to the model and all other parameters (intercepts, prognostic factor effects and residual variances) will be stratified by trial to account for the clustering of patients within studies. Maximum likelihood with quadrature will be used as estimation method and study-specific centering of the variables.(45) Variables potentially affecting the outcome that, despite randomisation, show baseline imbalances across treatment arms will be considered for adjustment based on the criteria for confounder selection by VanderWeele and Shpitser.(46-49) Procedure duration is considered an important proxy for the complexity of the procedure and will also be considered for adjustment despite being measured during the exposure.(48, 49) We assume that the FiO_2 used does not affect procedure duration. Benjamini-Hochberg correction will be used to account for multiple testing for the primary and secondary outcomes when appropriate.(50)

Exploration of variation in effects

For peer review only - http://bmjopen.bmj.com/site/about/guidelines.xhtml

To explore the causes of heterogeneity and identify factors modifying the effects of high intraoperative FiO_2, we will perform pre-specified subgroup analyses by extending the one-step meta-analysis framework to include treatment-covariate interaction terms. Subgroups will be defined according to mean core temperature (<35°C), mean net fluid supplementation (<15ml/kg/hr), use of mechanical ventilation, use of nitrous oxide, use of preoperative antibiotic prophylaxis, and procedure duration (>2.5h). All subgroup variables have been proposed as effect modifiers in previous studies and have a plausible biological substantiation.(51-62) Cut-offs are driven by previously reported data.(51-62) Treatment-covariate interaction terms $p < 0.05$ will be considered statistically significant. Dose-response variation will be explored by total O_2 exposure duration for each primary outcome. All exploratory analysis will be interpreted with caution considering the limited power and potential of type 1 error when multiple interactions are tested.

Additional analysis

A sensitivity analysis will be conducted to test the impact of excluding trials using N_2O as a carrier gas on the pooled effect estimate. Further, the choice of SSI definition will be evaluated in a sensitivity analysis applying the CDC definition as the primary outcome. In the case of exclusion of trials due to concerns on IPD integrity, a sensitivity analysis will be conducted to test the impact of including the concerning data on the pooled effect estimate. When multiple imputation is performed, a complete case analysis will also be conducted. In studies with sufficiently detailed data on the intervention, all analyses will also be conducted according to the per-protocol principle after adjustment for confounding factors due to incomplete adherence to the assigned treatments or use of off-protocol concomitant therapies according to the variable selection principles described for the primary analysis. Per protocol treatment will be defined as an FiO_2 of 0.80 ± 0.05 for at least 75% of the ventilation time in the intervention group, and an FiO_2 smaller than 0.40 with a margin of 0.05, for 75% in the control group. Patients requiring more oxygen for medical reasons, for example to maintain adequate saturation, after initial ventilation with an FiO_2 of 0.45 are exempted and not considered a protocol deviation. A sensitivity analysis will be conducted according to the two-step approach. All studies will be reanalysed separately, similarly to the one-step approach but without the term for trial clustering. The new aggregate data of each study will then be synthesised in a second step synthesising an overall estimate using maximum likelihood method followed by the Hartung-Knapp Sidik-Jonkman correction assuming random effects.(63) Between-study variance will be evaluated using τ^2; in addition, the Chi^2 test for heterogeneity will be performed with $p < 0.100$ considered statistically significant. In the unlikely event that IPD will not be made available,

For peer review only - http://bmjopen.bmj.com/site/about/guidelines.xhtml

For peer review only

aggregate study data will be included in the analyses during step two. Any unforeseen challenge during the analysis or choice that leads to discussion in the steering group that cannot be resolved by consensus will also be subjected to sensitivity analysis. To assess robustness of the time to event outcomes a survival curve will be compared to the univariable version of the Cox proportional hazards regression analysis.

Certainty of the cumulative estimate

The Grading of Recommendations Assessment Development and Evaluation (GRADE) working group methodology will be used to assess the overall quality of evidence for the following domains: risk of bias, unexplained inconsistency, indirectness, imprecision, publication bias, and magnitude of effect. Additional domains may be considered where appropriate. Optimal information size, defined as the number of participants needed for a single adequately powered trial, was calculated assuming a type-1 error (α) of 0.05, a type 2 error (β) of 0.2 and a relative risk reduction of 0.25.(64) If a confidence interval failed to exclude appreciable benefit or harm, defined as a relative risk reduction or increase of 0.25, the quality of evidence will be downgraded regardless of the optimal information size.(64) The overall certainty will be classified using four levels: high, moderate, low, and very low.(65)

Software

Results will be processed using R 4.0.4.

ETHICS AND DISSEMINATION

Ethical approval

Because this concerns a study on existing de-identified patient data, the medical research involving human subjects act does not apply and no formal medical ethics review is required.

Dissemination

This protocol and the results of this study will be submitted to a peer-reviewed medical journal regardless of the outcome. The protocol will be submitted before the data is gathered and analysed.

For peer review only - http://bmjopen.bmj.com/site/about/guidelines.xhtml

For peer review only

Competing interests

S.W.J. reports receipt of grants from Photonics in Healthcare, Integra LifeSciences, and Ethicon outside the submitted. P.S.M. reports receipt of grants or contracts from the Australian National Health and Medical Research Council (NHMRC), Practitioner Fellowship and Projects Grants, payment of expert testimony from Avant Medical Indemnity, and participation on a Data Safety Monitoring Board of Advisory Board for the SNAP, TOPIC-2 and BONANZA trials. A.F. reports receipt of institutional grants from the Australian Research Council Discovery Project and National Health and Medical Research Council Ideas outside the submitted work and participation on a Data Safety Monitoring Board or Advisory Board for the Australian Kidney Trials Research Network (INCH-HD, IMPEDE, TEQCH-PD, PHOSPHATE, BEST Fluids, N3RO trial, CKD-FIX, IMPROVE-FIX). R.G. reports participation on Steering Committee for the IntuBot Innosuisse Projekt and has a leadership role as ERC Director of Guidelines and ILCOR, and ILCOR Task Force Chair on Education, Implementation and Team, and Treasurer of European Airway Management society, and reports receipt without any payment of airway equipment for the research of the following: Intersurgical, Karl Storz, Verathon Inc, Aircraft Medical, Prodol Meditec, Venner Medical, Kingsystems, Medtronic, Ambu, VBM, Radiometer, Sentec, and Fisher & Paykel. A.K. reports receipt of grants or contracts from Potrero Medical, Rehabtronics and The 37company outside the submitted work and participation on a Data Safety Monitoring Board of Advisory Board in Directedsystems, Potrero Medical and BioAgel Laboratories. J.M. reports voluntary participation as a panellist in the updated WHO Guidelines on high vs low FiO2 in 2018 and voluntary co-investigator in the PENGUIN trial of high versus low FiO2 for SSI prevention in abdominal surgery in low- and middle-income settings with GlobalSurg Collaborative. M.G.W.D. reports participation on a Data Safety Monitoring Board or Advisory Board for the following trials: DANCE, SPHINX, ICONIC, SAFE, PACER, LEARNS, RECAP, and BIOPEX2. C.F. reports receipt fees for lectures and educational events from Getinge and Medtronic outside the submitted work. C.S. reports receipts of institutional grants from NICHD outside the submitted work. M.A.B. reports receipt of institutional grants from KCI/3M, Johnson & Johnson, New Compliance, BD Bard, Gore, Telabio, GDM, Medtronic, and Smith & Nephew outside the submitted work, and participation on the Data Monitoring Committee of the EXTEND trial. M.W.H. reports receipt of institutional grants from ZonMw outside the submitted work, consulting institutional fees from IDD Pharma outside the submitted work, institutional payment or honoraria for lectures, presentations, speakers bureaus, manuscript writing or educational events from CSL Behring outside the submitted work, and has a leadership role in DGAI, ISAP and IARS (Anaesthesia and Analgesia). The other authors declare no conflict of interest.

For peer review only - http://bmjopen.bmj.com/site/about/guidelines.xhtml

Funding

This work was supported by the Amsterdams Universiteitsfonds grant (4069) to R. H. Hulskes.

Contributions

SJ conceived the study. SJ, MH, MB and RH secured funding for the study. SJ, MH, MB, MD, and RH designed the study, drafted the study protocol and provided statistical expertise. RH and MZ provided input for the literature search and will coordinate the assembly of the data and perform the screening, inclusion and assessment of risk of bias. RW, CM, KL, PM, AF, RG, OA, AK, DS, JM, KP, JB, CF, GG, CS, DM, MC, PT, JO and BA provided substantial contributions to the study design, provided critical feedback and approved the final version of the study protocol. All authors compliant with their responsibilities according to the research protocol, meet authorship criteria as defined by the international committee of medical journal editors.

Acknowledgements

The authors thank Faridi Jamaludin as professional information specialist for the search.

Data sharing

Data will be made available upon reasonable request and review and approval of all collaborators.

For peer review only

For peer review only - http://bmjopen.bmj.com/site/about/guidelines.xhtml

References

1. Magill SS, Edwards JR, Bamberg W, Beldavs ZG, Dumyati G, Kainer MA, et al. Multistate point-prevalence survey of health care-associated infections. The New England journal of medicine. 2014;370(13):1198-208.
2. de Lissovoy G, Fraeman K, Hutchins V, Murphy D, Song D, Vaughn BB. Surgical site infection: incidence and impact on hospital utilization and treatment costs. American journal of infection control. 2009;37(5):387-97.
3. Control ECfDPa. Point prevalence survey of healthcareassociated

infections and antimicrobial use in European acute care hospitals. Stockholm: ECDC; 2013.
4. ECDC. Surveillance of surgical site infections in Europe 2010–2011. Stockholm: ECDC; 2013.
5. Leaper DJ, van Goor H, Reilly J, Petrosillo N, Geiss HK, Torres AJ, et al. Surgical site infection - a European perspective of incidence and economic burden. Int Wound J. 2004;1(4):247-73.
6. Koek MBG, van der Kooi TII, Stigter FCA, de Boer PT, de Gier B, Hopmans TEM, et al. Burden of surgical site infections in the Netherlands: cost analyses and disability-adjusted life years. J Hosp Infect. 2019;103(3):293-302.
7. Allegranzi B, Bischoff P, de Jonge S, Kubilay NZ, Zayed B, Gomes SM, et al. New WHO recommendations on preoperative measures for surgical site infection prevention: an evidence-based global perspective. Lancet Infect Dis. 2016;16(12):e276-e87.
8. Allegranzi B, Zayed B, Bischoff P, Kubilay NZ, de Jonge S, de Vries F, et al. New WHO recommendations on intraoperative and postoperative measures for surgical site infection prevention: an evidence-based global perspective. Lancet Infect Dis. 2016;16(12):e288-e303.
9. Berrios-Torres SI, Umscheid CA, Bratzler DW, Leas B, Stone EC, Kelz RR, et al. Centers for Disease Control and Prevention Guideline for the Prevention of Surgical Site Infection, 2017. JAMA Surg. 2017.
10. Hedenstierna G, Perchiazzi G, Meyhoff CS, Larsson A. Who Can Make Sense of the WHO Guidelines to Prevent Surgical Site Infection? Anesthesiology. 2017;126(5):771-3.
11. Volk T, Peters J, Sessler DI. The WHO recommendation for 80% perioperative oxygen is poorly justified. Anaesthesist. 2017;66(4):227-9.
12. Wenk M, Van Aken H, Zarbock A. The New World Health Organization Recommendations on Perioperative Administration of Oxygen to Prevent Surgical Site Infections: A Dangerous Reductionist Approach? Anesth Analg. 2017;125(2):682-7.
13. Meyhoff CS, Fonnes S, Wetterslev J, Jorgensen LN, Rasmussen LS. WHO Guidelines to prevent surgical site infections. Lancet Infect Dis. 2017;17(3):261-2.
14. Mellin-Olsen J, McDougall RJ, Cheng D. WHO Guidelines to prevent surgical site infections. Lancet Infect Dis. 2017;17(3):260-1.
15. Berrios-Torres SI, Umscheid CA, Bratzler DW, Leas B, Stone EC, Kelz RR, et al. Centers for Disease Control and Prevention Guideline for the Prevention of Surgical Site Infection, 2017. JAMA Surg. 2017;152(8):784-91.
16. Solomkin J, Egger M, de Jonge S, Latif A, Loke YK, Berenholtz S, et al. World Health Organization Responds to Concerns about Surgical Site Infection Prevention Recommendations. Anesthesiology. 2018;128(1):221-2.
17. Akca O, Ball L, Belda FJ, Biro P, Cortegiani A, Eden A, et al. WHO Needs High FIO2? Turk J Anaesthesiol Reanim. 2017;45(4):181-92.
18. Weenink RP, de Jonge SW, Preckel B, Hollmann MW. PRO: Routine hyperoxygenation in adult surgical patients whose tracheas are intubated. Anaesthesia. 2020;75(10):1293-6.
19. Sperna Weiland NH, Berger MM, Helmerhorst HJF. CON: Routine hyperoxygenation in adult surgical patients whose tracheas are intubated. Anaesthesia. 2020;75(10):1297-300.
20. Weenink RP, de Jonge SW, van Hulst RA, Wingelaar TT, van Ooij PAM, Immink RV, et al. Perioperative Hyperoxyphobia: Justified or Not? Benefits and Harms of Hyperoxia during Surgery. J Clin Med. 2020;9(3).

For peer review only - http://bmjopen.bmj.com/site/about/guidelines.xhtml

21. Mattishent K, Thavarajah M, Sinha A, Peel A, Egger M, Solomkin J, et al. Safety of 80% vs 30-35% fraction of inspired oxygen in patients undergoing surgery: a systematic review and meta-analysis. Br J Anaesth. 2019;122(3):311-24.
22. de Jonge S, Egger M, Latif A, Loke YK, Berenholtz S, Boermeester M, et al. Effectiveness of 80% vs 30-35% fraction of inspired oxygen in patients undergoing surgery: an updated systematic review and meta-analysis. Br J Anaesth. 2019;122(3):325-34.
23. Global Guidelines for the Prevention of Surgical Site Infection, (2018).
24. Staehr-Rye AK, Meyhoff CS, Scheffenbichler FT, Vidal Melo MF, Gätke MR, Walsh JL, et al. High intraoperative inspiratory oxygen fraction and risk of major respiratory complications. BJA: British Journal of Anaesthesia. 2017;119(1):140-9.
25. Greif R, Akça O, Horn EP, Kurz A, Sessler DI. Supplemental perioperative oxygen to reduce the incidence of surgical-wound infection. New England journal of medicine. 2000;342(3):161-7.
26. Kurz A, Kopyeva T, Suliman I, Podolyak A, You J, Lewis B, et al. Supplemental oxygen and surgical-site infections: an alternating intervention controlled trial. Br J Anaesth. 2018;120(1):117-26.
27. Kurz A, Sessler DI, Lenhardt R. Perioperative normothermia to reduce the incidence of surgical-wound infection and shorten hospitalization. Study of Wound Infection and Temperature Group. The New England journal of medicine. 1996;334(19):1209-15.
28. Atkinson TM, Giraud GD, Togioka BM, Jones DB, Cigarroa JE. Cardiovascular and Ventilatory Consequences of Laparoscopic Surgery. Circulation. 2017;135(7):700-10.
29. Doherty M, Buggy DJ. Intraoperative fluids: how much is too much? Br J Anaesth. 2012;109(1):69-79.
30. Sessler DI. Perioperative thermoregulation and heat balance. Lancet. 2016;387(10038):2655-64.
31. Riley RD, Lambert PC, Abo-Zaid G. Meta-analysis of individual participant data: rationale, conduct, and reporting. BMJ. 2010;340:c221.
32. Stewart LA, Clarke M, Rovers M, Riley RD, Simmonds M, Stewart G, et al. Preferred Reporting Items for Systematic Review and Meta-Analyses of individual participant data: the PRISMA-IPD Statement. JAMA. 2015;313(16):1657-65.
33. Oxman AD, Clarke MJ, Stewart LA. From science to practice. Meta-analyses using individual patient data are needed. JAMA. 1995;274(10):845-6.
34. Stewart LA, Parmar MK. Meta-analysis of the literature or of individual patient data: is there a difference? Lancet. 1993;341(8842):418-22.
35. Shamseer L, Moher D, Clarke M, Ghersi D, Liberati A, Petticrew M, et al. Preferred reporting items for systematic review and meta-analysis protocols (PRISMA-P) 2015: elaboration and explanation. BMJ. 2015;350:g7647.
36. SurfFilesender [SURFfilesender arose from the Filesender project (www.filesender.org). We developed SURFfilesender to meet the needs of Dutch education and research institutions. With this web application you can send any large file easily and securely.]. Available from: https://filesender.surf.nl.
37. Garner JS. CDC guideline for prevention of surgical wound infections, 1985. Supersedes guideline for prevention of surgical wound infections published in 1982. (Originally published in November 1985). Revised. Infect Control. 1986;7(3):193-200.
38. Culver DH, Horan TC, Gaynes RP, Martone WJ, Jarvis WR, Emori TG, et al. Surgical wound infection rates by wound class, operative procedure, and patient risk index. National Nosocomial Infections Surveillance System. Am J Med. 1991;91(3B):152S-7S.
39. van Walraven C, Musselman R. The Surgical Site Infection Risk Score (SSIRS): A Model to Predict the Risk of Surgical Site Infections. PLoS One. 2013;8(6):e67167.
40. INTEGRATED ADDENDUM TO ICH E6(R1): GUIDELINE FOR GOOD CLINICAL PRACTICE E6(R2), (2016).

For peer review only - http://bmjopen.bmj.com/site/about/guidelines.xhtml

41. Mangram AJ, Horan TC, Pearson ML, Silver LC, Jarvis WR. Guideline for Prevention of Surgical Site Infection, 1999. Centers for Disease Control and Prevention (CDC) Hospital Infection Control Practices Advisory Committee. American journal of infection control. 1999;27:97-132.
42. Rahbari NN, Weitz J, Hohenberger W, Heald RJ, Moran B, Ulrich A, et al. Definition and grading of anastomotic leakage following anterior resection of the rectum: a proposal by the International Study Group of Rectal Cancer. Surgery. 2010;147(3):339-51.
43. Sterne JAC, Savovic J, Page MJ, Elbers RG, Blencowe NS, Boutron I, et al. RoB 2: a revised tool for assessing risk of bias in randomised trials. BMJ. 2019;366:l4898.
44. Peters JL, Sutton AJ, Jones DR, Abrams KR, Rushton L. Contour-enhanced meta-analysis funnel plots help distinguish publication bias from other causes of asymmetry. J Clin Epidemiol. 2008;61(10):991-6.
45. Riley RD, Legha A, Jackson D, Morris TP, Ensor J, Snell KIE, et al. One-stage individual participant data meta-analysis models for continuous and binary outcomes: Comparison of treatment coding options and estimation methods. Statistics in Medicine. 2020;39(19):2536-55.
46. VanderWeele TJ. Principles of confounder selection. Eur J Epidemiol. 2019;34(3):211-9.
47. VanderWeele TJ, Shpitser I. A new criterion for confounder selection. Biometrics. 2011;67(4):1406-13.
48. Haley RW, Culver DH, Morgan WM, White JW, Emori TG, Hooton TM. Identifying patients at high risk of surgical wound infection. A simple multivariate index of patient susceptibility and wound contamination. Am J Epidemiol. 1985;121(2):206-15.
49. Korol E, Johnston K, Waser N, Sifakis F, Jafri HS, Lo M, et al. A systematic review of risk factors associated with surgical site infections among surgical patients. PLoS One. 2013;8(12):e83743.
50. Thissen D, Steinberg L, Kuang D. Quick and Easy Implementation of the Benjamini-Hochberg Procedure for Controlling the False Positive Rate in Multiple Comparisons. Journal of Educational and Behavioral Statistics. 2002;27(1):77-83.
51. Pryor KO, Fahey TJ, 3rd, Lien CA, Goldstein PA. Surgical site infection and the routine use of perioperative hyperoxia in a general surgical population: a randomized controlled trial. JAMA. 2004;291(1):79-87.
52. Belda FJ, Aguilera L, Garcia de la Asuncion J, Alberti J, Vicente R, Ferrandiz L, et al. Supplemental perioperative oxygen and the risk of surgical wound infection: a randomized controlled trial. JAMA. 2005;294(16):2035-42.
53. Meyhoff CS, Wetterslev J, Jorgensen LN, Henneberg SW, Hogdall C, Lundvall L, et al. Effect of high perioperative oxygen fraction on surgical site infection and pulmonary complications after abdominal surgery: the PROXI randomized clinical trial. JAMA. 2009;302(14):1543-50.
54. Bickel A, Gurevits M, Vamos R, Ivry S, Eitan A. Perioperative hyperoxygenation and wound site infection following surgery for acute appendicitis: a randomized, prospective, controlled trial. Arch Surg. 2011;146(4):464-70.
55. Duggal N, Poddatoori V, Noroozkhani S, Siddik-Ahmad RI, Caughey AB. Perioperative oxygen supplementation and surgical site infection after cesarean delivery: a randomized trial. Obstet Gynecol. 2013;122(1):79-84.
56. Gardella C, Goltra LB, Laschansky E, Drolette L, Magaret A, Chadwick HS, et al. High-concentration supplemental perioperative oxygen to reduce the incidence of postcesarean surgical site infection: a randomized controlled trial. Obstet Gynecol. 2008;112(3):545-52.
57. Mayzler O, Weksler N, Domchik S, Klein M, Mizrahi S, Gurman GM. Does supplemental perioperative oxygen administration reduce the incidence of wound infection in elective colorectal surgery? Minerva Anestesiol. 2005;71(1-2):21-5.
58. Myles PS, Leslie K, Chan MT, Forbes A, Paech MJ, Peyton P, et al. Avoidance of nitrous oxide for patients undergoing major surgery: a randomized controlled trial. Anesthesiology. 2007;107(2):221-31.
59. Stall A, Paryavi E, Gupta R, Zadnik M, Hui E, O'Toole RV. Perioperative supplemental oxygen to reduce surgical site infection after open fixation of high-risk fractures: a randomized controlled pilot trial. J Trauma Acute Care Surg. 2013;75(4):657-63.

For peer review only - http://bmjopen.bmj.com/site/about/guidelines.xhtml

60. Scifres CM, Leighton BL, Fogertey PJ, Macones GA, Stamilio DM. Supplemental oxygen for the prevention of postcesarean infectious morbidity: a randomized controlled trial. Am J Obstet Gynecol. 2011;205(3):267 e1-9.
61. Williams NL, Glover MM, Crisp C, Acton AL, McKenna DS. Randomized controlled trial of the effect of 30% versus 80% fraction of inspired oxygen on cesarean delivery surgical site infection. Am J Perinatol. 2013;30(9):781-6.
62. Thibon P, Borgey F, Boutreux S, Hanouz JL, Le Coutour X, Parienti JJ. Effect of perioperative oxygen supplementation on 30-day surgical site infection rate in abdominal, gynecologic, and breast surgery: the ISO2 randomized controlled trial. Anesthesiology. 2012;117(3):504-11.
63. Langan D, Higgins JPT, Jackson D, Bowden J, Veroniki AA, Kontopantelis E, et al. A comparison of heterogeneity variance estimators in simulated random-effects meta-analyses. Res Synth Methods. 2019;10(1):83-98.
64. Schünemann H, Brożek J, Guyatt G, Oxman A. Handbook for grading the quality of evidence and the strength of recommendations using the GRADE approach (updated October 2013). GRADE Working Group, 2013.
65. Guyatt GH, Oxman AD, Vist GE, Kunz R, Falck-Ytter Y, Alonso-Coello P, et al. GRADE: an emerging consensus on rating quality of evidence and strength of recommendations. BMJ. 2008;336(7650):924-6.

For peer review only - http://bmjopen.bmj.com/site/about/guidelines.xhtml

PRISMA-P (Preferred Reporting Items for Systematic review and Meta-Analysis Protocols) 2015 checklist: recommended items to address in a systematic review protocol*

Section and topic	Item No	Checklist item	Reported on page
**ADMINISTRATIVE INFORMATION**			
Title:			
Identification	1a	Identify the report as a protocol of a systematic review	1
Update	1b	If the protocol is for an update of a previous systematic review, identify as such	NA
Registration	2	If registered, provide the name of the registry (such as PROSPERO) and registration number	2
Authors:			
Contact	3a	Provide name, institutional affiliation, e-mail address of all protocol authors; provide physical mailing address of corresponding author	1
Contributions	3b	Describe contributions of protocol authors and identify the guarantor of the review	13
Amendments	4	If the protocol represents an amendment of a previously completed or published protocol, identify as such and list changes; otherwise, state plan for documenting important protocol amendments	6
Support:			
Sources	5a	Indicate sources of financial or other support for the review	13
Sponsor	5b	Provide name for the review funder and/or sponsor	13
Role of sponsor or funder	5c	Describe roles of funder(s), sponsor(s), and/or institution(s), if any, in developing the protocol	13
**INTRODUCTION**			
Rationale	6	Describe the rationale for the review in the context of what is already known	4-5
Objectives	7	Provide an explicit statement of the question(s) the review will address with reference to participants, interventions, comparators, and outcomes (PICO)	4
**METHODS**			
Eligibility criteria	8	Specify the study characteristics (such as PICO, study design, setting, time frame) and report characteristics (such as years considered, language, publication status) to be used as criteria for eligibility for the review	7
Information sources	9	Describe all intended information sources (such as electronic databases, contact with study authors, trial registers or other grey literature sources) with planned dates of coverage	7
Search strategy	10	Present draft of search strategy to be used for at least one electronic database, including planned limits, such that it could be repeated	Supplement01

For peer review only

Study records:			
Data management	11a	Describe the mechanism(s) that will be used to manage records and data throughout the review	8
Selection process	11b	State the process that will be used for selecting studies (such as two independent reviewers) through each phase of the review (that is, screening, eligibility and inclusion in meta-analysis)	7
Data collection process	11c any	Describe planned method of extracting data from reports (such as piloting forms, done independently, in duplicate), processes for obtaining and confirming data from investigators	8
Data items	12	List and define all variables for which data will be sought (such as PICO items, funding sources), any pre-planned data assumptions and simplifications	8-9
Outcomes and prioritization	13	List and define all outcomes for which data will be sought, including prioritization of main and additional outcomes, with rationale	9
Risk of bias in individual studies	14	Describe anticipated methods for assessing risk of bias of individual studies, including whether this will be done at the outcome or study level, or both; state how this information will be used in data synthesis	9-10
Data synthesis	15a	Describe criteria under which study data will be quantitatively synthesised	11
	15b	If data are appropriate for quantitative synthesis, describe planned summary measures, methods of handling data and methods of combining data from studies, including any planned exploration of consistency (such as I^2, Kendall's τ)	12
	15c	Describe any proposed additional analyses (such as sensitivity or subgroup analyses, meta-regression)	12
	15d	If quantitative synthesis is not appropriate, describe the type of summary planned	NA
Meta-bias(es)	16	Specify any planned assessment of meta-bias(es) (such as publication bias across studies, selective reporting within studies)	10
Confidence in cumulative evidence	17	Describe how the strength of the body of evidence will be assessed (such as GRADE)	12-13

*** It is strongly recommended that this checklist be read in conjunction with the PRISMA-P Explanation and Elaboration (cite when available) for important clarification on the items. Amendments to a review protocol should be tracked and dated. The copyright for PRISMA-P (including checklist) is held by the PRISMA-P Group and is distributed under a Creative Commons Attribution Licence 4.0.**

From: Shamseer L, Moher D, Clarke M, Ghersi D, Liberati A, Petticrew M, Shekelle P, Stewart L, PRISMA-P Group. Preferred reporting items for systematic review and meta-analysis protocols (PRISMA-P) 2015: elaboration and explanation. BMJ. 2015 Jan 2;349(jan02 1):g7647.

For peer review only

For peer review only - http://bmjopen.bmj.com/site/about/guidelines.xhtml

BMJ Open

The benefits and harms of perioperative high fraction inspired oxygen for surgical site infection prevention: a protocol for a systematic review and meta-analysis of individual patient data of randomised controlled trials

Journal:	*BMJ Open*
Manuscript ID	bmjopen-2022-067243.R2
Article Type:	Protocol
Date Submitted by the Author:	30-Jun-2023

Complete List of Authors:	de Jonge, Stijn; Amsterdam UMC Locatie AMC, Surgery; Amsterdam UMC Locatie AMC, Anaesthesiology<br>Hulskes, Rick; Amsterdam UMC Locatie AMC, Anaesthesiology; Amsterdam UMC Locatie AMC, Surgery<br>Zokaei Nikoo, Maedeh; Cleveland Clinic Foundation, Outcomes research<br>Weenink, Robert ; Amsterdam UMC Locatie AMC, Anaesthesiology<br>Meyhoff, Christian; Copenhagen University Hospital, Anaesthesia and Intensive Care<br>Leslie, Kate; The Royal Melbourne Hospital, Anaesthesia<br>Myles, Paul; Monash University, Anaesthesia and Perioperative Medicine<br>Forbes, Andrew; Monash University, Epidemiology and Preventive Medicine<br>Greif, Robert ; Inselspital Universitatsspital Bern, Anaesthesiology and PAin Therapy<br>Akca, Ozan; Johns Hopkins University, Anaesthesiology & Critical Care Medicine<br>Kurz, Andrea; Cleveland Clinic, General Anesthesiology<br>Sessler, Daniel; Cleveland Clinic,<br>Martin, Janet; University of Western Ontario, Anaesthesiology & Perioperative Medicine and Department of Epidemiology & Biostatistics<br>Dijkgraaf, Marcel; Amsterdam UMC Locatie AMC, Epidemiology and Data Science<br>Pryor, Kane; Weill Cornell Medical College, Anesthesiology<br>Belda, F. Javier; Hospital Clinico Universitario, Surgery; Hospital Clinico Universitario, Anaesthesia and Critical Care<br>Ferrando, Carlos; Hospital Clinico Universitario, Anesthesiology and Cirtical Care<br>Gurman, Gabriel; Ben-Gurion University of the Negev, Anaesthesiology and Critical Care Medicine<br>Scifres, Christina; Indiana University School of Medicine,<br>McKenna, David; Wright State University, Obstetrics and Gynaecology<br>Chan, MTV; The Chinese University of Hong Kong, Anesthesia and Intensive Care<br>Thibon, Pascal; Centre d'appui pour la prevention des infections associees aux soins d'Ile-de-France<br>Mellin-Olsen, Jannicke; World Federation of Societies of Anesthesiologists<br>Allegranzi, B; World Health Organization<br>Boermeester, Marja; Amsterdam UMC Locatie AMC, Surgery

	Hollmann, Markus; Amsterdam UMC Locatie AMC, Anaesthesiology
**Primary Subject Heading**:	Anaesthesia
Secondary Subject Heading:	Surgery
Keywords:	ANAESTHETICS, WOUND MANAGEMENT, Infection control < INFECTIOUS DISEASES, SURGERY

SCHOLARONE™
Manuscripts

TITLE PAGE

The benefits and harms of perioperative high fraction inspired oxygen for surgical site infection prevention: a protocol for a systematic review and meta-analysis of individual patient data of randomised controlled trials

S. W. de Jonge[1a,b,2*], R. H. Hulskes[1a,2], M. Zokaei Nikoo[11], R. P. Weenink[2], C. S. Meyhoff[3], K. Leslie[4], P. S. Myles[5], A. Forbes[6], R. Greif[7,8], O. Akça[9], A. Kurz[10], D. I. Sessler[11], J. Martin[12], M. G. Dijkgraaf[13a,b], K. O. Pryor[14], F. J. Belda[15], C. Ferrando[16a,16b], G. M. Gurman[17], C. Scifres[18], D. S. McKenna[19], M. T. V. Chan[20], P. Thibon[21], J. Mellin-Olsen[22], B. A. Allegranzi[23], M. A. Boermeester[1a, b], M. W. Hollmann[2]

[1a] Department of Surgery, Amsterdam UMC, University of Amsterdam, Amsterdam, The Netherlands
[1b] Gastroenterology Endocrinology & Metabolism, Amsterdam, the Netherlands, Amsterdam, The Netherlands
[2] Department of Anaesthesiology, Amsterdam UMC, University of Amsterdam, Amsterdam, The Netherlands
[3] Department of Anaesthesia and Intensive Care, Copenhagen University Hospital – Bispebjerg and Frederiksberg, Copenhagen, Denmark
[4] Department of Critical Care, Melbourne Medical School, University of Melbourne, Australia
[5] Department of Anaesthesiology and Perioperative Medicine, Alfred Hospital and Monash University, Melbourne, Australia
[6] Department of Epidemiology and Preventive Medicine, Monash University, Melbourne, Australia
[7] Department of Anaesthesiology and Pain Medicine, Bern University Hospital, University of Bern, Bern, Switzerland
[8] School of Medicine, Sigmund Freud University Vienna, Vienna, Austria
[9] Department of Anaesthesiology & Critical Care Medicine, Johns Hopkins University, Maryland, United States of America
[10] Department of Anaesthesiology and department of Outcomes Research, Cleveland Clinic Cleveland Ohio, Ohio, United States of America
[11] Department of Outcomes Research, Cleveland Clinic Cleveland Ohio, Ohio, United States of America
[12] Department of Anaesthesiology & Perioperative Medicine and Department of Epidemiology & Biostatistics, University of Western Ontario, Canada
[13a] Amsterdam UMC location University of Amsterdam, Epidemiology and Data Science, Amsterdam, The Netherlands
[13b] Amsterdam Public Health, Methodology, Amsterdam, The Netherlands
[14] Department of Anaesthesiology, Weil Medical College of Cornell University, New York City, New York, United States of America
[15] Department of Surgery and Department of Anaesthesia Critical Care, Hospital Clinico Universitario De Valencia, Valencia, Spain
[16a] Department of Anaesthesiology and Critical Care, Hospital Clínic I Provincial, Barcelona, Spain
[16b] CIBER de Enfermedades Respiratorias, Instituto de Salud Carlos III, Madrid, Spain
[17] Department of Anaesthesiology and Critical Care Medicine, Ben Gurion University of the Negev, Beer Sheva, Israel
[18] Department of Obstetrics and Gynaecology, Indiana University School of Medicine, Indianapolis, United States of America
[19] Department of Obstetrics and Gynaecology, Wright State University and Miami Valley Hospital, Ohio, United States of America
[20] Department of Anaesthesia and Intensive Care, Chinese University of Hong Kong, Hong Kong Special Administrative Region, China
[21] Centre d'appui pour la Prévention des Infections Associées aux Soins, CPias Normandie, Centre Hospitalo-Universitaire, Caen, Normandy, France
[22] World Federation of Societies of Anaesthesiologists
[23] World Health Organization, Service Delivery and Safety, Infection Prevention and Control Global Unit

***Corresponding author**

Corresponding author: S. W. de Jonge, Amsterdam UMC, University of Amsterdam, Meibergdreef 9, 1105 AZ Amsterdam, the Netherlands, email: s.w.dejonge@amsterdamumc.nl, telephone number 0031 (0)20 566 9111.

Word count: 2923 (excluding title page, abstract, article summary, tables, ethics and dissemination, and references)

For peer review only

For peer review only - http://bmjopen.bmj.com/site/about/guidelines.xhtml

46 **Keywords:** general anaesthesia; hyperoxia; postoperative outcome; surgical site infection; surgical wound infection

For peer review only

For peer review only - http://bmjopen.bmj.com/site/about/guidelines.xhtml

ABSTRACT

Introduction: The use of high fraction of inspired oxygen (FiO_2) intraoperatively for the prevention of surgical site infection (SSI) remains controversial. Promising results of early randomized controlled trials (RCT) have been replicated with varying success and subsequent meta-analysis are equivocal. Recent advancements in perioperative care, including the increased use of laparoscopic surgery and pneumoperitoneum and shifts in fluid and temperature management, can affect peripheral oxygen delivery and may explain the inconsistency in reproducibility. However, the published data provides insufficient detail on the participant level to test these hypotheses. The purpose of this individual participant data meta-analysis is to assess the described benefits and harms of intraoperative high FiO_2 compared to regular (0.21-0.40) FiO_2 and its potential effect modifiers.

Methods and analysis:

Two reviewers will search medical databases and online trial registries, including MEDLINE, Embase, CENTRAL, CINAHL, ClinicalTrials.gov, and WHO regional databases, for randomised and quasi randomised controlled trials comparing the effect of intraoperative high FiO2 (0.60-1.00) to regular FiO2 (0.21-0.40) on SSI within 90 days after surgery in adult patients. Secondary outcome will be all-cause mortality within the longest available follow-up. Investigators of the identified trials will be invited to collaborate. Data will be analysed with the one step approach using the generalised linear mixed model framework and the statistical model appropriate for the type of outcome being analysed (logistic and cox regression respectively), with a random treatment effect term to account for the clustering of patients within studies. The bias will be assessed using the RoB2 and the certainty of evidence using GRADE methodology. Pre-specified subgroup analyses include use of mechanical ventilation, nitrous oxide, preoperative antibiotic prophylaxis, temperature (<35°C), fluid supplementation (<15ml/kg/hr) and procedure duration (>2.5h).

Ethics and dissemination: Ethics approval is not required. Investigators will de-identify individual participant data before it is shared. The results will be submitted to a peer-review journal.

Trial Registration Number: PROSPERO CRD42018090261

For peer review only - http://bmjopen.bmj.com/site/about/guidelines.xhtml

ARTICLE SUMMARY

Strengths and limitations of this study

- Individual participant data meta-analysis (IPD MA) of (quasi-)randomised controlled trials provides the best possible analysis of the available data on the participant level, permitting the investigation of potential effect modifiers.
- IPD MA requires the collaboration of all investigators that have published data on the relevant topic and leads to a broad consensus on the outcome and interpretation of the analysis
- IPD MA depends on the quality of data that is made available by the authors of the original studies.

For peer review only

INTRODUCTION

Surgical site infection (SSI) is one of the most common healthcare-associated infections and leads to morbidity, mortality, and longer hospital stay.(1-4) The attributable costs can be more than €14.000 per SSI, and European totals are estimated to range from €1.5 to €19 billion per year.(5, 6) In 2016-2017, both the World Health Organization (WHO) and the Centres of Disease Control and Prevention (CDC) independently released evidence-based guidelines on the prevention of SSI that included a recommendation in favour of the administration of high fraction of inspired (FiO_2) for patients undergoing surgery under general anaesthesia.(7-9) This has led to a debate between opponents and proponents of the use of high FiO_2 in several editorials and correspondences across medical speciality literature.(10-20) Concerns were raised on the safety of the use of high FiO_2 as well as on the conflicting study results with some in support of the use of high FiO_2 to reduce SSI and some not.(10-20) Finally, studies by of one of the authors that contributed to the body of evidence were retracted because of unreproducible statistics.

In response to these concerns, the WHO conducted an independent systematic review on the safety of high intraoperative FiO_2 and updated the systematic review on its effectiveness, excluding the disputed trials.(21, 22) No evidence of harm to discourage the use of high FiO_2 was found, yet the evidence of an effect of SSI had become weaker, and the recommendation was adjusted accordingly.(23) Despite various studies and recommendations, there is still no consensus on the safety and effectiveness of using high FiO_2 during surgery with regard to SSI, all-cause mortality and other adverse events in adult patients. This leads to practice variation that inevitably exposes patients to suboptimal care.(24) There is a need for better understanding and consensus on this issue.

Since the early promising results, perioperative care has changed considerably. Open abdominal surgery has been largely replaced by laparoscopic surgery, fluid management has moved from liberal to restrictive, to advanced goal directed regimens and active perioperative warming has become a mainstay.(25-27) All these changes have considerable consequences for hemodynamic, microcirculation, and eventually peripheral oxygen delivery.(28-30) These changes may explain the inconsistency in reproducibility, but the available data provides insufficient detail on the participant level to test the potential of high FiO_2. Meta-analysis of individual participant data uses the raw individual-level data from the original study for synthesis and overcomes this limitation.(31, 32) IPD MA enables analysis of uniform outcomes with more statistical power and assessment of potential effect modifiers.(33, 34) Importantly, IPD MA requires collaboration with all published researchers on the topic leading to a broad consensus on the outcome of data analysis and interpretation.

For peer review only - http://bmjopen.bmj.com/site/about/guidelines.xhtml

The purpose of this IPD MA is to assess the potential benefits and harms of intraoperative high (0.60-1.00) FiO_2 compared to traditional (0.21-0.40) FiO_2 and its effect modifiers in adult patients undergoing surgery with SSI being the primary outcome. This IPD MA is initiated by the University of Amsterdam / Amsterdam University Medical Center, and encouraged by the WHO and the World Federation of Societies of Anaesthesiologists (WFSA) to provide patients and practitioners with the best possible evidence and guidance on this disputed area and will give clearance of the disputed hypothesis that high FiO_2 reduces the incidence of SSI.

For peer review only

For peer review only - http://bmjopen.bmj.com/site/about/guidelines.xhtml

METHODS AND ANALYSIS

Protocol and registration

This study protocol is registered with the International Prospective Register of Systematic Reviews (PROSPERO) on 7 March 2018 and was last updated on 15 July 2022 (registration number CRD42018090261). The study protocol is designed and written to adhere to the Preferred Reporting Items for Systematic Review and Meta-Analysis Protocols (PRISMA-P)(35) and the Preferred Reporting Items for Systematic Review Meta-Analysis of individual patient data (IPD) (PRISMA-IPD).(32)

Patient and Public Involvement Statement

This project is encouraged by the World Health Organization (WHO), and the World Federation of Societies of Anesthesiologists (WFSA) to provide patients and practitioners with the best possible evidence and guidance on this disputed area. WHO and WFSA have provided external independent review and advice on research direction and aim.

Governance

This study is an initiative of the Amsterdam University Medical Centre, encouraged by the WHO and the WFSA. Both organisations recognise the urgent need for this research and provide external independent review and advice. The writing committee consists of the study coordinator, two reviewers, a lead methodologist, and a principal investigator from both the surgery and the anaesthesiology department of the Amsterdam University Medical Centre and two external content matter experts. The writing committee is entirely independent of the initial trials and has full responsibility for all methodological decisions. A broader steering committee with representatives of the collaborating trial groups identified during the project will be invited to comment on and co-author the final protocol and IPD MA report. By sharing their IPD, collaborators will obtain one co-authorship on the IPD MA report and one additional co-authorship if data of more than 300 participants is shared. For transparency and against intellectual bias, a record will be kept of all comments. Any important amendments to the protocol will be recorded in PROSPERO record and discussed in the methods section of the final report.

Eligibility criteria

For peer review only - http://bmjopen.bmj.com/site/about/guidelines.xhtml

For peer review only

We will include all randomised and quasi-randomised controlled trials comparing the effect of intraoperative high FiO_2 (0.60-1.0) to traditional FiO_2 (0.21-0.40) in patients undergoing surgery. Definitions for high and low FiO_2 were determined by literature review and consensus among the IPDMA collaborators.(21, 22) These trials may include patients of any age undergoing surgery except for neonates, regardless publication, language, or year of conduct and should include at least data on age, sex, mean FiO_2 administered, method of oxygen administration, SSI, mortality, or other serious adverse events. Any outcome found to be recorded in these trials will be included in the analysis. Studies without random or quasi-random treatment allocation, animal studies, and studies outside of the intraoperative period will be excluded.

For peer review only

Identifying studies – information sources

The initial search conducted for the WHO guideline will be updated by a professional information specialist.(21, 22) Medical databases will be searched, including MEDLINE, Embase, CENTRAL, CINAHL, ClinicalTrials.gov, and the WHO regional databases. Online trial registries will be searched to identify potential unpublished evidence or any ongoing trials. The search will not be limited by language or date of publication. A final update will be conducted before the final round of revisions preceding submission for publication. The reference list of all included studies will be hand searched for any additional relevant trials not already identified through database searching. All corresponding authors of relevant clinical trials will be contacted to review the list of identified studies for the omission of potentially relevant studies missed by the search.

Study selection process

Two reviewers will independently assess articles retrieved by the search against the eligibility criteria. After screening the title and abstract using Rayyan, the full text of potentially eligible papers will be retrieved and assessed.(36) When no full paper exists, or trial eligibility is in doubt, the study authors will be contacted to provide further information. Any discrepancies in study selection will be resolved through consensus and discussion with a senior author. All studies that pass title and abstract screening but were not eligible for inclusion will be listed with the reasons for exclusion.

Study collaboration invitation

Authors of eligible studies will be contacted and invited to collaborate on the IPD MA. An email invitation will be sent to the corresponding authors outlining the IPMDA goals. If no reply is received

For peer review only - http://bmjopen.bmj.com/site/about/guidelines.xhtml

within two weeks, a second email request will be sent to the corresponding and first author. If no response is received again, we will try to contact all authors by email and telephone. IPD data will be considered unavailable if numerous times (at least five) no reply is received if authors no longer have access to the study data or consent to collaboration.

Data collection process

The collaborating investigators will be requested to sign a data transfer agreement describing the ownership and storage of the IPD before IPD is shared. Whenever possible, data collection, interview on the protocol, and formal handoff on the data codebook will be done electronically via email, videoconference, or a suitable alternative. Whenever requested by the original investigator, a researcher will visit the investigators for a physical data transfer, in-person interview, and data codebook handoff. IPD will be de-identified by the suppling collaborator. The IPD de-identification code will not be shared. IPD will be transferred using one of the following secure methods: SurfFilesender, a secure password protected data transfer service,(37) end-to-end encrypted and password protected using email or send by courier on a physical storage media. Once transferred, IPD will be stored securely on the local server of the Amsterdam UMC where appropriate data and privacy policies will be maintained, as well as procedures and associated physical, technical and administrative safeguards to assure that the IPD are accessed only by authorized personnel. In the unlikely event that individual patient data will not be made available, the reason will be recorded. The aggregate data of the study will be used in a sensitivity analysis. Aggregate data collection will be performed as appropriate for a regular meta-analysis by two independent reviewers according to a predefined data extraction sheet and overseen by a senior author to settle potential discrepancies. The University of Amsterdam's Clinical Research Unit will facilitate secure data storage.

For peer review only

Data items

Data items will include all data recorded by the initial trial investigators including, but not limited to the items listed in table 1 and table 2. SSI within 90 days after surgery according to the authors discretion will be the primary outcome, all-cause mortality within the longest available follow up will be the secondary outcome. All other outcomes are exploratory.

**Table 1. Baseline and procedure characteristics**	
Baseline	Sex, age, BMI (kg/m^2), ASA physical status score, smoking status, peripheral vascular disease, diabetes, (metastatic) cancer, congestive heart disease, (pulmonary) hypertension, chronic obstructive pulmonary disease,

For peer review only - http://bmjopen.bmj.com/site/about/guidelines.xhtml

	immunosuppressant use, peripheral oxygen saturation (%), glucose (mg/dL), indication for surgery, emergency procedure
Preoperative	Use of preoperative antibiotic prophylaxis (dose and agent), timing of preoperative antibiotic prophylaxis, use of mechanical bowel preparation, haemoglobin (g/dL), use of antibiotic bowel preparation, cytostatic chemotherapy, radiotherapy, Use of preoperative skin preparation prophylaxis (dose and agent), Timing of preoperative skin preparation prophylaxis.
Intraoperative	Surgical procedure(s), organ involvement, contamination (CDC wound classification(38)), laparoscopic surgery, mean arterial pressure (MAP) (mmHg), haemoglobin (g/dL), heart rate (beats/min), hemodynamic monitoring method, hemodynamic management algorithm, crystalloid infusion (ml), colloid Infusion (ml), red-cell transfusion (units), duration of surgery (min), duration of anaesthesia (min), mean core temperature (°C)*, lowest core temperature (°C)*, duration hypothermia (<35°C), mean net fluid supplementation (ml/kg/hr), arterial oxygen saturation (%), peripheral oxygen saturation (%), subcutaneous oxygen tension (mm Hg), muscle oxygen tension (mm Hg), partial pressure of arterial oxygen (mmHg), mean FiO_2 (%), mean PEEP (cmH_2O), ventilator flow (L/min), peak airway pressure (mmHg), plateau pressure (mmHg), tidal Volume (ml/kg predicted body weight), respiratory frequency, vasopressor agent, vasopressor agent used (mg), glucose (mg/dL), use of general anaesthesia, use of spinal or epidural anaesthesia, use of mechanical ventilation, use of nitrous oxide, total blood loss (ml), fluids (ml), end tidal CO_2
Postoperative	Use of postoperative antibiotics (dose and agent), postoperative antibiotic duration (days), cytostatic chemotherapy, radiotherapy, haemoglobin (g/dL), VAS pain score, use of postoperative oxygen suppletion (duration, method and FiO_2), haemoglobin (g/dL), peripheral oxygen saturation (%), partial pressure of arterial oxygen (mmHg), subcutaneous oxygen tension (mmHg), muscle oxygen tension (mmHg), glucose (mg/dL), NNIS score(39), SSI risk score(40), Use of drains
Oxygen Administration & Monitoring	Total duration and concentration of oxygen exposure during the pre/intra/postoperative period (timing of initiation, concentration, duration), oxygen supply and mode of administration (Intubation, use and type of face mask, nasal prongs), carrier gas (N_2, N_20, medical or room air), protocol-defined target or range of partial pressure of arterial oxygen (mmHg) or peripheral oxygen saturation (%)
* Direct measurement or its approximation by peripheral measurement	

219

Table 2. Outcome data and effect measure specification	
Primary	- SSI within 90 days after surgery by the author's discretion
Secondary	- All-cause mortality within the longest available follow-up
Exploratory	- Survival within the longest available follow-up<br>- Serious adverse events defined by the ICH guidelines for good clinical practice(41)<br>- SSI monitored according to the CDC criteria and specified as either superficial, deep, organ/space(42)<br>- Respiratory insufficiency: defined as the need for respiratory assistance provided as ventilator therapy or non-invasive ventilation within 90 days after surgery<br>- Unplanned ICU admission (not part of routine postoperative care) (days)

For peer review only - http://bmjopen.bmj.com/site/about/guidelines.xhtml

	- Hospital readmissions within 90 days after surgery<br>- Anastomotic leakage as defined by the international study group of rectal cancer(43)<br>- Total duration of hospitalization, including readmissions related to the initial hospitalization<br>- Any cardiovascular complication at any time after surgery<br>- Any pulmonary complications at any time after surgery<br>- Stroke at any time after surgery<br>- New or recurrent cancer diagnosis at any time after surgery<br>- Any further clinically relevant outcome reported in the IPD
* When patients are reoperated within follow up for reasons other than surgical site infection these cases will be excluded from the analysis based on loss to follow up.	

Missing data

When variables are missing at the participant level and the missing at random assumption is plausible, multiple imputations by chained equations may be applied in each trial separately before proceeding with the analysis. Variables that miss systematically i.e., unknown for the entire study or are deemed missing non-randomly after discussion in the writing committee, will not be imputated. When this concerns variables included in pre-defined analysis, studies systematically missing this variable will be excluded from that analysis. When this concerns variables not included in pre-defined analysis, these variables will be dropped from the main outcome analysis as potential confounding variables. The set of available variables for the main analysis will thus be determined by the data set with the least available variables. Variables from richer sets will remain available for exploratory analysis among data sets with the variable available.

Individual Participant Data integrity

We will check IPD for potential missing, invalid, or out-of-range values, inconsistencies, and discrepancies with the aggregate publication. When identified, we will seek to resolve the issues with the trial investigators to improve data quality and ensure that trials are represented accurately. In addition, any modelling assumptions made in the initial analysis will be evaluated (i.e., missing at random in the case of multiple imputation or non-informative censoring and proportional hazards in the case of time to event data). In the case of any concerns on IPD integrity further prove of execution of the trial and substantiation of the results may be requested such as prove of institutional review board approval or original case record forms. If concerns cannot be resolved with the trial investigators, the data of the concerning study will not be included in the primary analysis and the reason for exclusion will be explicitly stated.

For peer review only - http://bmjopen.bmj.com/site/about/guidelines.xhtml

Risk of bias

Two reviewers will independently assess the quality of the included studies using the Cochrane risk-of-bias tool for randomised trials (RoB2).(44) Studies will be judged as "*low risk", "some concerns", or "high risk* of bias". Publication bias will be assessed using a contour enhanced funnel plot.(45) Additionally, the IPD will be used to directly check process parameters of some of the bias domains. Randomization and allocation concealment will be assessed by checking baseline imbalances. Incomplete outcome data will be assessed by checking the IPD to ensure all randomized patients are included. All available clinically relevant outcomes in the IPD will be reported in the IPD MA. For time to event outcomes such as mortality, pattern and extent of follow up will be checked. When needed, additional follow up with original authors will be conducted to rectify any imbalances as far as possible.

Synthesis methods

All outcomes will be analysed according to the intention to treat principle and using a one-step approach for IPD MA. In the one-step approach, IPD will be modelled from all studies simultaneously using the generalised linear mixed model framework and the statistical model appropriate for the type of outcome being analysed (i.e., logistic regression for binary outcome data, linear regression for continuous outcome data, and Cox-regression for time to event data). A random treatment effect term will be added to the model and all other parameters (intercepts, prognostic factor effects and residual variances) will be stratified by trial to account for the clustering of patients within studies. Maximum likelihood with quadrature will be used as estimation method and study-specific centering of the variables.(46) Variables potentially affecting the outcome that, despite randomisation, show baseline imbalances across treatment arms will be considered for adjustment based on the criteria for confounder selection by VanderWeele and Shpitser.(47-50) Procedure duration is considered an important proxy for the complexity of the procedure and will also be considered for adjustment despite being measured during the exposure.(49, 50) We assume that the FiO_2 used does not affect procedure duration. Benjamini-Hochberg correction will be used to account for multiple testing for the primary and secondary outcomes when appropriate.(51)

Exploration of variation in effects

For peer review only - http://bmjopen.bmj.com/site/about/guidelines.xhtml

To explore the causes of heterogeneity and identify factors modifying the effects of high intraoperative FiO_2, we will perform pre-specified subgroup analyses by extending the one-step meta-analysis framework to include treatment-covariate interaction terms. Subgroups will be defined according to mean core temperature (<35°C), mean net fluid supplementation (<15ml/kg/hr), use of mechanical ventilation, use of nitrous oxide, use of preoperative antibiotic prophylaxis, and procedure duration (>2.5h). All subgroup variables have been proposed as effect modifiers in previous studies and have a plausible biological substantiation.(52-63) Cut-offs are driven by previously reported data.(52-63) Treatment-covariate interaction terms $p < 0.05$ will be considered statistically significant. Dose-response variation will be explored by total O_2 exposure duration for each primary outcome. All exploratory analysis will be interpreted with caution considering the limited power and potential of type 1 error when multiple interactions are tested.

Additional analysis

A sensitivity analysis will be conducted to test the impact of excluding trials using N_2O as a carrier gas on the pooled effect estimate. Further, the choice of SSI definition will be evaluated in a sensitivity analysis applying the CDC definition as the primary outcome. In the case of exclusion of trials due to concerns on IPD integrity, a sensitivity analysis will be conducted to test the impact of including the concerning data on the pooled effect estimate. When multiple imputation is performed, a complete case analysis will also be conducted. In studies with sufficiently detailed data on the intervention, all analyses will also be conducted according to the per-protocol principle after adjustment for confounding factors due to incomplete adherence to the assigned treatments or use of off-protocol concomitant therapies according to the variable selection principles described for the primary analysis. Per protocol treatment will be defined as an FiO_2 of 0.80 ± 0.05 for at least 75% of the ventilation time in the intervention group, and an FiO_2 smaller than 0.40 with a margin of 0.05, for 75% in the control group. Patients requiring more oxygen for medical reasons, for example to maintain adequate saturation, after initial ventilation with an FiO_2 of 0.45 are exempted and not considered a protocol deviation. A sensitivity analysis will be conducted according to the two-step approach. All studies will be reanalysed separately, similarly to the one-step approach but without the term for trial clustering. The new aggregate data of each study will then be synthesised in a second step synthesising an overall estimate using maximum likelihood method followed by the Hartung-Knapp Sidik-Jonkman correction assuming random effects.(64) Between-study variance will be evaluated using τ^2; in addition, the Chi^2 test for heterogeneity will be performed with $p < 0.100$ considered statistically significant. In the unlikely event that IPD will not be made available,

For peer review only - http://bmjopen.bmj.com/site/about/guidelines.xhtml

aggregate study data will be included in the analyses during step two. Any unforeseen challenge during the analysis or choice that leads to discussion in the steering group that cannot be resolved by consensus will also be subjected to sensitivity analysis. To assess robustness of the time to event outcomes a survival curve will be compared to the univariable version of the Cox proportional hazards regression analysis.

Certainty of the cumulative estimate

The Grading of Recommendations Assessment Development and Evaluation (GRADE) working group methodology will be used to assess the overall quality of evidence for the following domains: risk of bias, unexplained inconsistency, indirectness, imprecision, publication bias, and magnitude of effect. Additional domains may be considered where appropriate. Optimal information size, defined as the number of participants needed for a single adequately powered trial, was calculated assuming a type-1 error (α) of 0.05, a type 2 error (β) of 0.2 and a relative risk reduction of 0.25.(65) If a confidence interval failed to exclude appreciable benefit or harm, defined as a relative risk reduction or increase of 0.25, the quality of evidence will be downgraded regardless of the optimal information size.(65) The overall certainty will be classified using four levels: high, moderate, low, and very low.(66)

Software

Results will be processed using R 4.0.4.

ETHICS AND DISSEMINATION

Ethical approval

Because this concerns a study on existing de-identified patient data, the medical research involving human subjects act does not apply and no formal medical ethics review is required.

Dissemination

This protocol and the results of this study will be submitted to a peer-reviewed medical journal regardless of the outcome. The protocol will be submitted before the data is gathered and analysed.

For peer review only - http://bmjopen.bmj.com/site/about/guidelines.xhtml

Competing interests

S.W.J. reports receipt of grants from Photonics in Healthcare, Integra LifeSciences, and Ethicon outside the submitted. P.S.M. reports receipt of grants or contracts from the Australian National Health and Medical Research Council (NHMRC), Practitioner Fellowship and Projects Grants, payment of expert testimony from Avant Medical Indemnity, and participation on a Data Safety Monitoring Board of Advisory Board for the SNAP, TOPIC-2 and BONANZA trials. A.F. reports receipt of institutional grants from the Australian Research Council Discovery Project and National Health and Medical Research Council Ideas outside the submitted work and participation on a Data Safety Monitoring Board or Advisory Board for the Australian Kidney Trials Research Network (INCH-HD, IMPEDE, TEQCH-PD, PHOSPHATE, BEST Fluids, N3RO trial, CKD-FIX, IMPROVE-FIX). R.G. reports participation on Steering Committee for the IntuBot Innosuisse Projekt and has a leadership role as ERC Director of Guidelines and ILCOR, and ILCOR Task Force Chair on Education, Implementation and Team, and Treasurer of European Airway Management society, and reports receipt without any payment of airway equipment for the research of the following: Intersurgical, Karl Storz, Verathon Inc, Aircraft Medical, Prodol Meditec, Venner Medical, Kingsystems, Medtronic, Ambu, VBM, Radiometer, Sentec, and Fisher & Paykel. A.K. reports receipt of grants or contracts from Potrero Medical, Rehabtronics and The 37company outside the submitted work and participation on a Data Safety Monitoring Board of Advisory Board in Directedsystems, Potrero Medical and BioAgel Laboratories. J.M. reports voluntary participation as a panellist in the updated WHO Guidelines on high vs low FiO2 in 2018 and voluntary co-investigator in the PENGUIN trial of high versus low FiO2 for SSI prevention in abdominal surgery in low- and middle-income settings with GlobalSurg Collaborative. M.G.W.D. reports participation on a Data Safety Monitoring Board or Advisory Board for the following trials: DANCE, SPHINX, ICONIC, SAFE, PACER, LEARNS, RECAP, and BIOPEX2. C.F. reports receipt fees for lectures and educational events from Getinge and Medtronic outside the submitted work. C.S. reports receipts of institutional grants from NICHD outside the submitted work. M.A.B. reports receipt of institutional grants from KCI/3M, Johnson & Johnson, New Compliance, BD Bard, Gore, Telabio, GDM, Medtronic, and Smith & Nephew outside the submitted work, and participation on the Data Monitoring Committee of the EXTEND trial. M.W.H. reports receipt of institutional grants from ZonMw outside the submitted work, consulting institutional fees from IDD Pharma outside the submitted work, institutional payment or honoraria for lectures, presentations, speakers bureaus, manuscript writing or educational events from CSL Behring outside the submitted work, and has a leadership role in DGAI, ISAP and IARS (Anaesthesia and Analgesia). The other authors declare no conflict of interest.

For peer review only - http://bmjopen.bmj.com/site/about/guidelines.xhtml

Funding

This work was supported by the Amsterdams Universiteitsfonds grant (4069) to R. H. Hulskes.

Contributions

SJ conceived the study. SJ, MH, MB and RH secured funding for the study. SJ, MH, MB, MD, and RH designed the study, drafted the study protocol and provided statistical expertise. RH and MZ provided input for the literature search and will coordinate the assembly of the data and perform the screening, inclusion and assessment of risk of bias. RW, CM, KL, PM, AF, RG, OA, AK, DS, JM, KP, JB, CF, GG, CS, DM, MC, PT, JO and BA provided substantial contributions to the study design, provided critical feedback and approved the final version of the study protocol. All authors compliant with their responsibilities according to the research protocol, meet authorship criteria as defined by the international committee of medical journal editors.

Acknowledgements

The authors thank Faridi Jamaludin as professional information specialist for the search.

Data sharing

Data will be made available upon reasonable request and review and approval of all collaborators.

For peer review only

For peer review only - http://bmjopen.bmj.com/site/about/guidelines.xhtml

References

1. Magill SS, Edwards JR, Bamberg W, Beldavs ZG, Dumyati G, Kainer MA, et al. Multistate point-prevalence survey of health care-associated infections. N Engl J Med. 2014;370(13):1198-208.
2. de Lissovoy G, Fraeman K, Hutchins V, Murphy D, Song D, Vaughn BB. Surgical site infection: incidence and impact on hospital utilization and treatment costs. Am J Infect Control. 2009;37(5):387-97.
3. Control ECfDPa. Point prevalence survey of healthcareassociated infections and antimicrobial use in European acute care hospitals. Stockholm: ECDC; 2013.
4. ECDC. Surveillance of surgical site infections in Europe 2010–2011. Stockholm: ECDC; 2013.
5. Leaper DJ, van Goor H, Reilly J, Petrosillo N, Geiss HK, Torres AJ, et al. Surgical site infection - a European perspective of incidence and economic burden. Int Wound J. 2004;1(4):247-73.
6. Koek MBG, van der Kooi TII, Stigter FCA, de Boer PT, de Gier B, Hopmans TEM, et al. Burden of surgical site infections in the Netherlands: cost analyses and disability-adjusted life years. J Hosp Infect. 2019;103(3):293-302.
7. Allegranzi B, Bischoff P, de Jonge S, Kubilay NZ, Zayed B, Gomes SM, et al. New WHO recommendations on preoperative measures for surgical site infection prevention: an evidence-based global perspective. Lancet Infect Dis. 2016;16(12):e276-e87.
8. Allegranzi B, Zayed B, Bischoff P, Kubilay NZ, de Jonge S, de Vries F, et al. New WHO recommendations on intraoperative and postoperative measures for surgical site infection prevention: an evidence-based global perspective. Lancet Infect Dis. 2016;16(12):e288-e303.
9. Berrios-Torres SI, Umscheid CA, Bratzler DW, Leas B, Stone EC, Kelz RR, et al. Centers for Disease Control and Prevention Guideline for the Prevention of Surgical Site Infection, 2017. JAMA Surg. 2017.
10. Hedenstierna G, Perchiazzi G, Meyhoff CS, Larsson A. Who Can Make Sense of the WHO Guidelines to Prevent Surgical Site Infection? Anesthesiology. 2017;126(5):771-3.
11. Volk T, Peters J, Sessler DI. The WHO recommendation for 80% perioperative oxygen is poorly justified. Anaesthesist. 2017;66(4):227-9.
12. Wenk M, Van Aken H, Zarbock A. The New World Health Organization Recommendations on Perioperative Administration of Oxygen to Prevent Surgical Site Infections: A Dangerous Reductionist Approach? Anesth Analg. 2017;125(2):682-7.
13. Meyhoff CS, Fonnes S, Wetterslev J, Jorgensen LN, Rasmussen LS. WHO Guidelines to prevent surgical site infections. Lancet Infect Dis. 2017;17(3):261-2.
14. Mellin-Olsen J, McDougall RJ, Cheng D. WHO Guidelines to prevent surgical site infections. Lancet Infect Dis. 2017;17(3):260-1.
15. Berrios-Torres SI, Umscheid CA, Bratzler DW, Leas B, Stone EC, Kelz RR, et al. Centers for Disease Control and Prevention Guideline for the Prevention of Surgical Site Infection, 2017. JAMA Surg. 2017;152(8):784-91.
16. Solomkin J, Egger M, de Jonge S, Latif A, Loke YK, Berenholtz S, et al. World Health Organization Responds to Concerns about Surgical Site Infection Prevention Recommendations. Anesthesiology. 2018;128(1):221-2.
17. Akca O, Ball L, Belda FJ, Biro P, Cortegiani A, Eden A, et al. WHO Needs High FIO2? Turk J Anaesthesiol Reanim. 2017;45(4):181-92.
18. Weenink RP, de Jonge SW, Preckel B, Hollmann MW. PRO: Routine hyperoxygenation in adult surgical patients whose tracheas are intubated. Anaesthesia. 2020;75(10):1293-6.
19. Sperna Weiland NH, Berger MM, Helmerhorst HJF. CON: Routine hyperoxygenation in adult surgical patients whose tracheas are intubated. Anaesthesia. 2020;75(10):1297-300.
20. Weenink RP, de Jonge SW, van Hulst RA, Wingelaar TT, van Ooij PAM, Immink RV, et al. Perioperative Hyperoxyphobia: Justified or Not? Benefits and Harms of Hyperoxia during Surgery. J Clin Med. 2020;9(3).

For peer review only

For peer review only - http://bmjopen.bmj.com/site/about/guidelines.xhtml

21. Mattishent K, Thavarajah M, Sinha A, Peel A, Egger M, Solomkin J, et al. Safety of 80% vs 30-35% fraction of inspired oxygen in patients undergoing surgery: a systematic review and meta-analysis. Br J Anaesth. 2019;122(3):311-24.
22. de Jonge S, Egger M, Latif A, Loke YK, Berenholtz S, Boermeester M, et al. Effectiveness of 80% vs 30-35% fraction of inspired oxygen in patients undergoing surgery: an updated systematic review and meta-analysis. Br J Anaesth. 2019;122(3):325-34.
23. Global Guidelines for the Prevention of Surgical Site Infection, (2018).
24. Staehr-Rye AK, Meyhoff CS, Scheffenbichler FT, Vidal Melo MF, Gätke MR, Walsh JL, et al. High intraoperative inspiratory oxygen fraction and risk of major respiratory complications. BJA: British Journal of Anaesthesia. 2017;119(1):140-9.
25. Greif R, Akça O, Horn EP, Kurz A, Sessler DI. Supplemental perioperative oxygen to reduce the incidence of surgical-wound infection. New England journal of medicine. 2000;342(3):161-7.
26. Kurz A, Kopyeva T, Suliman I, Podolyak A, You J, Lewis B, et al. Supplemental oxygen and surgical-site infections: an alternating intervention controlled trial. Br J Anaesth. 2018;120(1):117-26.
27. Kurz A, Sessler DI, Lenhardt R. Perioperative normothermia to reduce the incidence of surgical-wound infection and shorten hospitalization. Study of Wound Infection and Temperature Group. N Engl J Med. 1996;334(19):1209-15.
28. Atkinson TM, Giraud GD, Togioka BM, Jones DB, Cigarroa JE. Cardiovascular and Ventilatory Consequences of Laparoscopic Surgery. Circulation. 2017;135(7):700-10.
29. Doherty M, Buggy DJ. Intraoperative fluids: how much is too much? Br J Anaesth. 2012;109(1):69-79.
30. Sessler DI. Perioperative thermoregulation and heat balance. Lancet. 2016;387(10038):2655-64.
31. Riley RD, Lambert PC, Abo-Zaid G. Meta-analysis of individual participant data: rationale, conduct, and reporting. BMJ. 2010;340:c221.
32. Stewart LA, Clarke M, Rovers M, Riley RD, Simmonds M, Stewart G, et al. Preferred Reporting Items for Systematic Review and Meta-Analyses of individual participant data: the PRISMA-IPD Statement. JAMA. 2015;313(16):1657-65.
33. Oxman AD, Clarke MJ, Stewart LA. From science to practice. Meta-analyses using individual patient data are needed. JAMA. 1995;274(10):845-6.
34. Stewart LA, Parmar MK. Meta-analysis of the literature or of individual patient data: is there a difference? Lancet. 1993;341(8842):418-22.
35. Shamseer L, Moher D, Clarke M, Ghersi D, Liberati A, Petticrew M, et al. Preferred reporting items for systematic review and meta-analysis protocols (PRISMA-P) 2015: elaboration and explanation. BMJ. 2015;350:g7647.
36. Rayyan---a web and mobile app for systematic reviews. Systematic Reviews. 2016;5(1):210.
37. SurfFilesender [SURFfilesender arose from the Filesender project (www.filesender.org). We developed SURFfilesender to meet the needs of Dutch education and research institutions. With this web application you can send any large file easily and securely.]. Available from: https://filesender.surf.nl.
38. Garner JS. CDC guideline for prevention of surgical wound infections, 1985. Supersedes guideline for prevention of surgical wound infections published in 1982. (Originally published in November 1985). Revised. Infect Control. 1986;7(3):193-200.
39. Culver DH, Horan TC, Gaynes RP, Martone WJ, Jarvis WR, Emori TG, et al. Surgical wound infection rates by wound class, operative procedure, and patient risk index. National Nosocomial Infections Surveillance System. Am J Med. 1991;91(3B):152S-7S.
40. van Walraven C, Musselman R. The Surgical Site Infection Risk Score (SSIRS): A Model to Predict the Risk of Surgical Site Infections. PLoS One. 2013;8(6):e67167.
41. INTEGRATED ADDENDUM TO ICH E6(R1): GUIDELINE FOR GOOD CLINICAL PRACTICE E6(R2), (2016).

For peer review only - http://bmjopen.bmj.com/site/about/guidelines.xhtml

42. Mangram AJ, Horan TC, Pearson ML, Silver LC, Jarvis WR. Guideline for Prevention of Surgical Site Infection, 1999. Centers for Disease Control and Prevention (CDC) Hospital Infection Control Practices Advisory Committee. Am J Infect Control. 1999;27:97-132.

43. Rahbari NN, Weitz J, Hohenberger W, Heald RJ, Moran B, Ulrich A, et al. Definition and grading of anastomotic leakage following anterior resection of the rectum: a proposal by the International Study Group of Rectal Cancer. Surgery. 2010;147(3):339-51.

44. Sterne JAC, Savovic J, Page MJ, Elbers RG, Blencowe NS, Boutron I, et al. RoB 2: a revised tool for assessing risk of bias in randomised trials. BMJ. 2019;366:l4898.

45. Peters JL, Sutton AJ, Jones DR, Abrams KR, Rushton L. Contour-enhanced meta-analysis funnel plots help distinguish publication bias from other causes of asymmetry. J Clin Epidemiol. 2008;61(10):991-6.

46. Riley RD, Legha A, Jackson D, Morris TP, Ensor J, Snell KIE, et al. One-stage individual participant data meta-analysis models for continuous and binary outcomes: Comparison of treatment coding options and estimation methods. Statistics in Medicine. 2020;39(19):2536-55.

47. VanderWeele TJ. Principles of confounder selection. Eur J Epidemiol. 2019;34(3):211-9.

48. VanderWeele TJ, Shpitser I. A new criterion for confounder selection. Biometrics. 2011;67(4):1406-13.

49. Haley RW, Culver DH, Morgan WM, White JW, Emori TG, Hooton TM. Identifying patients at high risk of surgical wound infection. A simple multivariate index of patient susceptibility and wound contamination. Am J Epidemiol. 1985;121(2):206-15.

50. Korol E, Johnston K, Waser N, Sifakis F, Jafri HS, Lo M, et al. A systematic review of risk factors associated with surgical site infections among surgical patients. PLoS One. 2013;8(12):e83743.

51. Thissen D, Steinberg L, Kuang D. Quick and Easy Implementation of the Benjamini-Hochberg Procedure for Controlling the False Positive Rate in Multiple Comparisons. Journal of Educational and Behavioral Statistics. 2002;27(1):77-83.

52. Pryor KO, Fahey TJ, 3rd, Lien CA, Goldstein PA. Surgical site infection and the routine use of perioperative hyperoxia in a general surgical population: a randomized controlled trial. JAMA. 2004;291(1):79-87.

53. Belda FJ, Aguilera L, Garcia de la Asuncion J, Alberti J, Vicente R, Ferrandiz L, et al. Supplemental perioperative oxygen and the risk of surgical wound infection: a randomized controlled trial. JAMA. 2005;294(16):2035-42.

54. Meyhoff CS, Wetterslev J, Jorgensen LN, Henneberg SW, Hogdall C, Lundvall L, et al. Effect of high perioperative oxygen fraction on surgical site infection and pulmonary complications after abdominal surgery: the PROXI randomized clinical trial. JAMA. 2009;302(14):1543-50.

55. Bickel A, Gurevits M, Vamos R, Ivry S, Eitan A. Perioperative hyperoxygenation and wound site infection following surgery for acute appendicitis: a randomized, prospective, controlled trial. Arch Surg. 2011;146(4):464-70.

56. Duggal N, Poddatoori V, Noroozkhani S, Siddik-Ahmad RI, Caughey AB. Perioperative oxygen supplementation and surgical site infection after cesarean delivery: a randomized trial. Obstet Gynecol. 2013;122(1):79-84.

57. Gardella C, Goltra LB, Laschansky E, Drolette L, Magaret A, Chadwick HS, et al. High-concentration supplemental perioperative oxygen to reduce the incidence of postcesarean surgical site infection: a randomized controlled trial. Obstet Gynecol. 2008;112(3):545-52.

58. Mayzler O, Weksler N, Domchik S, Klein M, Mizrahi S, Gurman GM. Does supplemental perioperative oxygen administration reduce the incidence of wound infection in elective colorectal surgery? Minerva Anestesiol. 2005;71(1-2):21-5.

59. Myles PS, Leslie K, Chan MT, Forbes A, Paech MJ, Peyton P, et al. Avoidance of nitrous oxide for patients undergoing major surgery: a randomized controlled trial. Anesthesiology. 2007;107(2):221-31.

60. Stall A, Paryavi E, Gupta R, Zadnik M, Hui E, O'Toole RV. Perioperative supplemental oxygen to reduce surgical site infection after open fixation of high-risk fractures: a randomized controlled pilot trial. J Trauma Acute Care Surg. 2013;75(4):657-63.

For peer review only

For peer review only - http://bmjopen.bmj.com/site/about/guidelines.xhtml

61. Scifres CM, Leighton BL, Fogertey PJ, Macones GA, Stamilio DM. Supplemental oxygen for the prevention of postcesarean infectious morbidity: a randomized controlled trial. Am J Obstet Gynecol. 2011;205(3):267 e1-9.
62. Williams NL, Glover MM, Crisp C, Acton AL, McKenna DS. Randomized controlled trial of the effect of 30% versus 80% fraction of inspired oxygen on cesarean delivery surgical site infection. Am J Perinatol. 2013;30(9):781-6.
63. Thibon P, Borgey F, Boutreux S, Hanouz JL, Le Coutour X, Parienti JJ. Effect of perioperative oxygen supplementation on 30-day surgical site infection rate in abdominal, gynecologic, and breast surgery: the ISO2 randomized controlled trial. Anesthesiology. 2012;117(3):504-11.
64. Langan D, Higgins JPT, Jackson D, Bowden J, Veroniki AA, Kontopantelis E, et al. A comparison of heterogeneity variance estimators in simulated random-effects meta-analyses. Res Synth Methods. 2019;10(1):83-98.
65. Schünemann H, Brożek J, Guyatt G, Oxman A. Handbook for grading the quality of evidence and the strength of recommendations using the GRADE approach (updated October 2013). GRADE Working Group, 2013.
66. Guyatt GH, Oxman AD, Vist GE, Kunz R, Falck-Ytter Y, Alonso-Coello P, et al. GRADE: an emerging consensus on rating quality of evidence and strength of recommendations. BMJ. 2008;336(7650):924-6.

For peer review only

PRISMA-P (Preferred Reporting Items for Systematic review and Meta-Analysis Protocols) 2015 checklist: recommended items to address in a systematic review protocol*

Section and topic	Item No	Checklist item	Reported on page
**ADMINISTRATIVE INFORMATION**			
Title:			
Identification	1a	Identify the report as a protocol of a systematic review	1
Update	1b	If the protocol is for an update of a previous systematic review, identify as such	NA
Registration	2	If registered, provide the name of the registry (such as PROSPERO) and registration number	2
Authors:			
Contact	3a	Provide name, institutional affiliation, e-mail address of all protocol authors; provide physical mailing address of corresponding author	1
Contributions	3b	Describe contributions of protocol authors and identify the guarantor of the review	13
Amendments	4	If the protocol represents an amendment of a previously completed or published protocol, identify as such and list changes; otherwise, state plan for documenting important protocol amendments	6
Support:			
Sources	5a	Indicate sources of financial or other support for the review	13
Sponsor	5b	Provide name for the review funder and/or sponsor	13
Role of sponsor or funder	5c	Describe roles of funder(s), sponsor(s), and/or institution(s), if any, in developing the protocol	13
**INTRODUCTION**			
Rationale	6	Describe the rationale for the review in the context of what is already known	4-5
Objectives	7	Provide an explicit statement of the question(s) the review will address with reference to participants, interventions, comparators, and outcomes (PICO)	4
**METHODS**			
Eligibility criteria	8	Specify the study characteristics (such as PICO, study design, setting, time frame) and report characteristics (such as years considered, language, publication status) to be used as criteria for eligibility for the review	7
Information sources	9	Describe all intended information sources (such as electronic databases, contact with study authors, trial registers or other grey literature sources) with planned dates of coverage	7
Search strategy	10	Present draft of search strategy to be used for at least one electronic database, including planned limits, such that it could be repeated	Supplement01

For peer review only

Study records:			
Data management	11a	Describe the mechanism(s) that will be used to manage records and data throughout the review	8
Selection process	11b	State the process that will be used for selecting studies (such as two independent reviewers) through each phase of the review (that is, screening, eligibility and inclusion in meta-analysis)	7
Data collection process	11c any	Describe planned method of extracting data from reports (such as piloting forms, done independently, in duplicate), processes for obtaining and confirming data from investigators	8
Data items	12	List and define all variables for which data will be sought (such as PICO items, funding sources), any pre-planned data assumptions and simplifications	8-9
Outcomes and prioritization	13	List and define all outcomes for which data will be sought, including prioritization of main and additional outcomes, with rationale	9
Risk of bias in individual studies	14	Describe anticipated methods for assessing risk of bias of individual studies, including whether this will be done at the outcome or study level, or both; state how this information will be used in data synthesis	9-10
Data synthesis	15a	Describe criteria under which study data will be quantitatively synthesised	11
	15b	If data are appropriate for quantitative synthesis, describe planned summary measures, methods of handling data and methods of combining data from studies, including any planned exploration of consistency (such as I^2, Kendall's τ)	12
	15c	Describe any proposed additional analyses (such as sensitivity or subgroup analyses, meta-regression)	12
	15d	If quantitative synthesis is not appropriate, describe the type of summary planned	NA
Meta-bias(es)	16	Specify any planned assessment of meta-bias(es) (such as publication bias across studies, selective reporting within studies)	10
Confidence in cumulative evidence	17	Describe how the strength of the body of evidence will be assessed (such as GRADE)	12-13

*** It is strongly recommended that this checklist be read in conjunction with the PRISMA-P Explanation and Elaboration (cite when available) for important clarification on the items. Amendments to a review protocol should be tracked and dated. The copyright for PRISMA-P (including checklist) is held by the PRISMA-P Group and is distributed under a Creative Commons Attribution Licence 4.0.**

From: Shamseer L, Moher D, Clarke M, Ghersi D, Liberati A, Petticrew M, Shekelle P, Stewart L, PRISMA-P Group. Preferred reporting items for systematic review and meta-analysis protocols (PRISMA-P) 2015: elaboration and explanation. BMJ. 2015 Jan 2;349(jan02 1):g7647.

For peer review only - http://bmjopen.bmj.com/site/about/guidelines.xhtml
